# From patterned response dependency to structured covariate dependency: Entropy based categorical-pattern-matching

**DOI:** 10.1371/journal.pone.0198253

**Published:** 2018-06-14

**Authors:** Hsieh Fushing, Shan-Yu Liu, Yin-Chen Hsieh, Brenda McCowan

**Affiliations:** 1 Department of Statistics, University of California Davis, Davis, California, United States of America; 2 Department of Computer Science, University of California Davis, Davis, California, United States of America; 3 School of Veterinary Medicine, University of California Davis, Davis, California, United States of America; University of Virginia, UNITED STATES

## Abstract

Data generated from a system of interest typically consists of measurements on many covariate features and possibly multiple response features across all subjects in a designated ensemble. Such data is naturally represented by one response-matrix against one covariate-matrix. A matrix lattice is an advantageous platform for simultaneously accommodating heterogeneous data types: continuous, discrete and categorical, and exploring hidden dependency among/between features and subjects. After each feature being individually renormalized with respect to its own histogram, the categorical version of mutual conditional entropy is evaluated for all pairs of response and covariate features according to the combinatorial information theory. Then, by applying Data Could Geometry (DCG) algorithmic computations on such a mutual conditional entropy matrix, multiple synergistic feature-groups are partitioned. Distinct synergistic feature-groups embrace distinct structures of dependency. The explicit details of dependency among members of synergistic features are seen through mutliscale compositions of blocks computed by a computing paradigm called Data Mechanics. We then propose a categorical pattern matching approach to establish a directed associative linkage: from the patterned response dependency to serial structured covariate dependency. The graphic display of such a directed associative linkage is termed an information flow and the degrees of association are evaluated via tree-to-tree mutual conditional entropy. This new universal way of discovering system knowledge is illustrated through five data sets. In each case, the emergent visible heterogeneity is an organization of discovered knowledge.

## Introduction

Nearly all scientific researches are geared to acquire knowledge and understanding on systems of interest. So data generated from a target system typically consists of measurements on many covariate features and possibly multiple response features belonging to subjects, who constitute a representative ensemble of the system. As such a system data set typically consists of one response data matrix against a covariate data matrix. These two matrices share the common ensemble of subjects, which are arranged along its row-axis, while their own features are arranged along their own column-axis, respectively. The matrix lattice is indeed an advantageous platform for revealing patterned structures, particularly for dependency within response or covariate sides. Moreover these two platforms become the joint foundation for all the directed associative linkages going from response side to covariate side.

It is known that, among these subjects, whether they are human, animal or plants or even cells, are likely interconnected, and among these relevant features, no matter they are on either response or covariate sides, are interrelated. Such interconnections and interrelatedness also weave interacting relations between subjects and features. Thus, as a rule, each system data set is expected to contain these three fronts of dependency, which have unknown detailed structures, and wait to be explored and discovered. Further we conceive the system understanding and knowledge as the linkages going from response’s dependency structures to covariate’s dependency constructs.

A guideline for successfully extracting system understanding and knowledge was indeed laid more than four decades ago in physics. The Physics Nobel laureate P. W. Anderson [1], in his Science paper with title “More is different”, has pointed out that the task of “synthesis” upon a complex system is all but impossible. Given that almost all systems of scientific interests are complex, any endeavor of data analysis in line with the task of synthesis, such as any supervised version of learning or modeling, is likely futile in gaining system understanding or knowledge. Therefore, a truly beneficial system data analysis should embrace a protocol that build upon strategies by giving up the concept of “synthesis via modeling” completely.

In this paper we propose a protocol for analyzing any system data set. A quick overview of this protocol is given as follows. First, we adopt unsupervised data-driven computing, which is free from any unrealistic structural or distributional assumptions, in order to effectively capture authentic information of dependency structures and constructs. Secondly, we employ a graphic display platform to arrange and represent extracted information in order to stimulate understanding and explore knowledge regarding the system under study. The guiding principle underlying such a graphic display platform is appealing to the formidable capability of visual processing in man [2].

Our protocol embraces a simple, but distinctive concept: a system likely contains multiple system mechanisms in both response and covariate sides. To embrace this concept in a natural fashion, a nonlinear association measure is evaluated upon each pair of features on the response and covariate sides. We then apply an Ultrametric clustering algorithm to partition the collection of covariate features into a composition of synergistic feature clusters (or groups). Likewise the collection of response features are partitioned. Each synergistic feature group functionally reveals a distinctive pattern of dependency, so indeed represents a distinct mechanism of the target system. Therefore, we need to seek for interpretation pertaining to a single response mechanism through its linkages to a series of covariate mechanisms. A computational algorithm coupled with a graphic display platform is developed to make such linkages explicitly interpretable and pictorially visible. It is worth emphasizing that system understanding and knowledge involved with multiple mechanisms in multiple different ways. Also it is evident to note that this composite-level concept fundamentally and contrastingly distinguishes this data analysis protocol from statistical modeling and supervised learning.

In contrast, traditional statistical modeling and popular supervised learning share three common key characteristics: 1) primarily accommodate only one single response feature at a time, which destroys the entire response dependency; 2) utilizes conditioning argument on covariate features, which ignores covariate dependency and potential involvements of multiple mechanisms; 3) and imposes independence among subjects, which imposes unrealistic homogeneity. At the end, data-analysis results are pushed through the likelihood principle or an optimizing process with respect to a man-made criterion.

Further model-based techniques only accommodate selective data types, in others words, a data type often becomes the decisive factor in choosing models. For a binary response feature, the logistic regression model is the definite choice. For a continuous response feature, the linear regression is the definite choice. The logistic and linear modeling frameworks break down when the response feature has more two categories. When two or more response features are of interest, statistical modeling break down as well. The latter, so-called multiple response issue, was raised more than half century by John Tukey [3]. Up to today there exist no satisfactory solutions in literature. At this era of big data, aforementioned shortcomings of statistical modeling and supervised learning certainly would be exposed further and wider than ever before.

Current state of lacking a universal platform for building directed associative linkages from response side to covariate side needs to be changed. In this paper we envision that our protocol for system data analysis embraces such a universal platform, which can accommodate all data-types and multiple response features. The computational developments of this protocol begin with a rather unconventional approach: **basically re-normalizing all features into categorical ones sharing a common digital-coding range**. A real-valued feature is re-normalized into a digital-categorical via its own possibly-gapped histogram [4]. We believe that digital-categorical is the most fundamental data type. This categorical nature makes possible for employing combinatorial information theory to define the mutual conditional entropy, which is the most basic and reliable evaluation of possibly nonlinear nonsymmetric association. Since it basically relies only on counting. Unlike the linear correlation, this entropy-based nonlinear association is meaningful by having no hypothetical assumptions.

Also it is equally important to note that the purpose of such a re-normalization is to make all features digitally comparable. This comparability paves a valid foundation for computing and representing structural dependency via similarity on response and covariate sides. That is, upon a matrix constituted by a group of renormalized synergistic features arranged along the column axis against all subjects along its row axis, the computing for structural dependency is primarily performed through the application of Data Mechanics (DM), an unsupervised learning algorithm developed in [5] and [6]. Data Mechanics algorithm merely carries out permutations on row- and column-axes of such a matrix in order to achieve the nearly minimum total-variation, or energy, on the matrix-lattice. This tremendous amount of computations for the minimization task was surprisingly achieved by iteratively applying the Data Cloud Geometry (DCG), an Utrametric clustering algorithm developed in [7] and [8], to build one clustering tree upon subjects on its row axis and another clustering tree upon features on its column axis.

When these two marginal clustering trees resulted from the DM computations are superimposed respectively on the two axes of the matrix, visible multiscale patterned-blocks emerge on the permuted matrix lattice, which is consequently termed heatmap. This heatmap collectively reveals a detailed version of mechanism-specific structural dependency by showing the three fronts of information contents contained in the data matrix: 1) how and why some subjects group closely together, and how and why they are far away from other clusters of subjects; 2) how and why the Ultrametric tree on features represents and constitute a map of key factors of the system; 3) how and why the multiscale block-patterns bring out the heterogeneity in a collective fashion through the interacting relational characteristics between subject and features.

We further construct a directed associative linkage from one heatmap pertaining to a response mechanism to another heatmap pertaining to a covariate mechanism. The construction via a graphic display exhibits that such a linkage maps the memberships of a clustering composition taken from the response’s clustering tree on subjects (row-axis) onto a clustering composition taken from the covariate’s clustering tree on subjects. That is, based on the two trees, each subject is encoded with a pair of categorical code: one from response side and the other from the covariate side. Therefore, the strength of such a linkage is again evaluated by the directed conditional-entropy based on the combinatorial information theory. This graphic display in fact facilitates the interpretation of the linkage by matching multiscale block-patterns of the response mechanism to the multiscale block-patterns of the covariate mechanism. So the interpretation is explicit, visible and readable. This graphic linkage is called categorical-pattern matching. We then extend this platform of graphic display to accommodate a series of a covariate mechanisms, and term it an Information flow.

## Materials and methods: Conceptual and computational foundations in data analysis

**Evaluation of amount of information conveyed by *X* with regard to *Y*.** In his 1965 paper [9] with title “Three approaches to the quantitative definition of information,” A. N. Kolmogorov said that

“‥ It is only important for me to show that the mathematical problems associated with a purely combinatorial approach to the measure of information are not limited to trivialities.”

Indeed the combinatorial approach has been well known in Information Theory since C. E. Shannon’s pioneer works [10] [11]. However, outside of Communication and Information Theories, its use in real world data analysis is not yet evident, neither popular nor widespread. This is not because it is not useful, but rather because it has been overshadowed by the concept of “correlation” in Statistics, which is nothing but an inner product of two unit vectors in mathematics without the rigorous checking on the bivariate Normality assumption.

In this paper we discuss that the combinatorial approach of information in fact gives us an universal measure of associative relation between two variables. Based on conditional entropy, this relational association concept will be seen as especially suitable for unsupervised machine learning and its inferences, in which the “sample-to-population” sense is not involved. Along the developing process, we also reflect why the linearity backbone of correlation can cause invalid and even often misleading interpretations on real data. Before introducing such a measure of entropy based associative relation, it is beneficial to review this combinatorial approach of Information.

Consider and denote the amount of uncertainty, say $A\left(N\right)=H\left(\frac{1}{N},.....,\frac{1}{N}\right)$, of choosing one subject with uniformly equal potentials among *N*(= *m* × *n*) subjects contained within an ensemble. If these *N* subjects are divided in *m* sub-ensembles of size *n*, then the equal-potential sampling scheme on *N* subjects is equivalent to first sampling with equal potentials from the collection of *m* sub-ensembles, and secondly sampling one subject with equal potentials from the chosen sub-ensemble of *n* subjects. This so called composition rule [12], implies that the uncertainty *A*(*N*) = *A*(*n* × *m*) = *A*(*m*) + *A*(*n*). Shannon has determined that such *A*(*N*) = *C* × *logN* up to a constant *C* [10]. Let’s choose a *C*, such that

$$\begin{array}{c} A\left(N\right)=-\sum_{1}^{N}\log_{2}\frac{1}{N}=\log_{2}N. \end{array}$$

In general, if *N* subjects are marked by numbers and partitioned in *K* color-coded sub-ensembles possibly unequal sizes (*N*_1_, …*N*_*K*_), that is, two variables are defined upon this ensemble of *N* subjects: let the variable *Y* be the number-coding from 1 to *N*, and *X* be the color-coding. Let the *K* color-coded sub-ensembles have their proportion being denoted as ${\left\{p_{k}=\frac{N_{k}}{N}\right\}}_{k=1}^{K}$, then the entropy of discrete variable *X* pertaining to sampling with probability ${\left\{P_{k}\right\}}_{k=1}^{K}$ is generically calculated and denoted as

$$\begin{array}{c} H\left(X\right)=-\sum_{k=1}^{K}p_{k}\log p_{k}=A\left(N\right)-\sum_{k=1}^{K}p_{k}A\left(N_{k}\right). \end{array}$$

This equation says that the amount of information conveyed by variable *X* with regard to the variable *Y*, say *I*[*Y*: *X*], is exactly equal to *H*(*X*) [9]. Here we use the notation for the above equation as

$$\begin{array}{c} E\left[Y\to X\right]=H\left(Y\right)-H\left(Y|X\right)=H\left(Y\right)-\sum_{k=1}^{K}p_{k}H\left(Y|X=k\right). \end{array}$$

Based on such combinatorial information theory, the mutual conditional-entropy for two clustering compositions is pictorially illustrated in S1 Box of Supporting Information, while their formulas are contained in S2 Box. Specifically the tree at bottom of S1 Box is a subset of X-covariate tree for *Y*(color coding) given *X* = *a* and its corresponding conditional entropy is developed at the beginning of S2 Box.

**Factors and mechanisms in a system of interest.** When a system is under study, it is universal that many dimensions of feature-specific measurements are observed or measured from subjects, which are constituents of the system. Unless they are coordinated according to temporal, spatial or other known axes, these features are typically unorganized with respect to a known framework. Nonetheless, just as coordinated features likely manifest evolving system states along the axes, these unorganized features also likely comprised of various distinct mechanisms of dependency. As such, system states and mechanisms as system’s major components are popularly called factors in many scientific fields, especially in psychology and economics and many other social sciences.

The popularity of factor analysis is based more on computational convenience than on meaningful interpretations [3]. Specifically these factors are conveniently computed via principle component analysis (PCA), singular value decomposition (SVD) and their dynamic variants based on covariance matrices. Hence, factor analysis is mainly used as a way of achieving linearity based dimension reduction and retaining major proportion of information contents under normality. However, since intricate patterns of dependency among features potentially go far beyond dyadic correlations, such factor analysis often incurs information loss and unnatural representations of underlying system mechanisms. That was partly why John Tukey strongly discouraged applications of factor analysis [3].

In order to naturally and explicitly reveal system mechanisms, it becomes necessary to demonstrate structural dependency among all features included in the data. Given that distinct mechanisms involve with distinct feature-groups of different sizes, the issue of how to re-group features to show distinct mechanisms becomes a pressing issue in any system study. One universal concept of dependency considered here is based on *E*[*Y* → *X*] and *E*[*X* → *Y*] of two features denoted by *X* and *Y*, that is, if *X* is capable of conveying a non-negligible amount of information in relation to *Y*, or vice versa, then *X* and *Y* are dependent. After building a mutual conditional-entropy matrix, subsequently, as will be demonstrated in sections blow, an unsupervised learning algorithm is applied to perform the task of feature regrouping. That is, a synergistic group of features is seen as constituting a mechanism, while two synergistic groups being antagonistic would be seen as two separate mechanisms. Unlike the logistic and linear regression models in statistics, our proposed computational protocol will link one synergistic response-feature group to one or several synergistic covariate-feature groups. This proposal interestingly fulfills Tukey’s postulation of appealing to Taxonomy and classification methodologies on the multiple response issue [3].

**System knowledge** In a system study, the primary goal of data analysis is to compute and organize visible knowledge pertaining to the linkages from a response’s mechanism to covariate’s mechanisms. Since all system’s mechanisms on both sides share the common space of constituent subjects belonging to the system under study. The linkages are supposed to be seen and built through this common space of subjects.

To be more specific, the simplest, but most important form of system knowledge linkage is prescribed with **heterogeneity** as: One serial uniform pattern-blocks framed by a serial synergistic covariate-feature groups and a cluster of subjects nearly exclusively explain one part of one whole pattern-block framed by a synergistic response-feature group and a **larger** cluster of subjects.

Here a block-pattern is taken as a knowledge locus, and a linkage via the exclusiveness is meant to be equipped with an extreme conditional entropy (*E*[*Y* → *X*]) of being near zero. Via this heterogeneity, scientists specifically figure out how the measurements of a synergistic response-feature group upon a subject-cluster can be explained collectively by multiple distinct series of block-patterns manifested by serial synergistic covariate-feature groups upon a partition of the original subject-cluster. From this perspective, our data analysis is clearly fundamentally distinct from convention statistical modeling and supervised learning methods, which heavily rely on the hypothesized sample-to-population homogeneity.

Unsupervised learning paradigms and combinatorial approach, as will discussed in the Method section, are ideal for computing and discovering knowledge representations and achieving the constructions of such linkages. In fact this computational paradigm and representational approach provide a means to safe guard against such apparent dangers of man-made inconsistency and fallacy. That is, system knowledge derived from this approach will necessarily reveal patterns of multiple scales that are realistically available from data.

## Computing methods

**Motivations and goals** Before introducing our computational paradigms for extracting information contained in a system data set, we motivate our developments by explaining why modeling methodologies in statistics have limited merits in many system studies. Here we use the popular Logistic regression as an illustrating example. We explicitly demonstrate why this modeling is not expandable mathematically, that is, this modeling is constrained strictly by the underlying homogeneity assumption, which goes against the heterogeneity naturally embedded within almost all systems of scientific interest. It is worth emphasizing that similar explanations would be applicable to the linear regression model as well.

**Fig 1:**
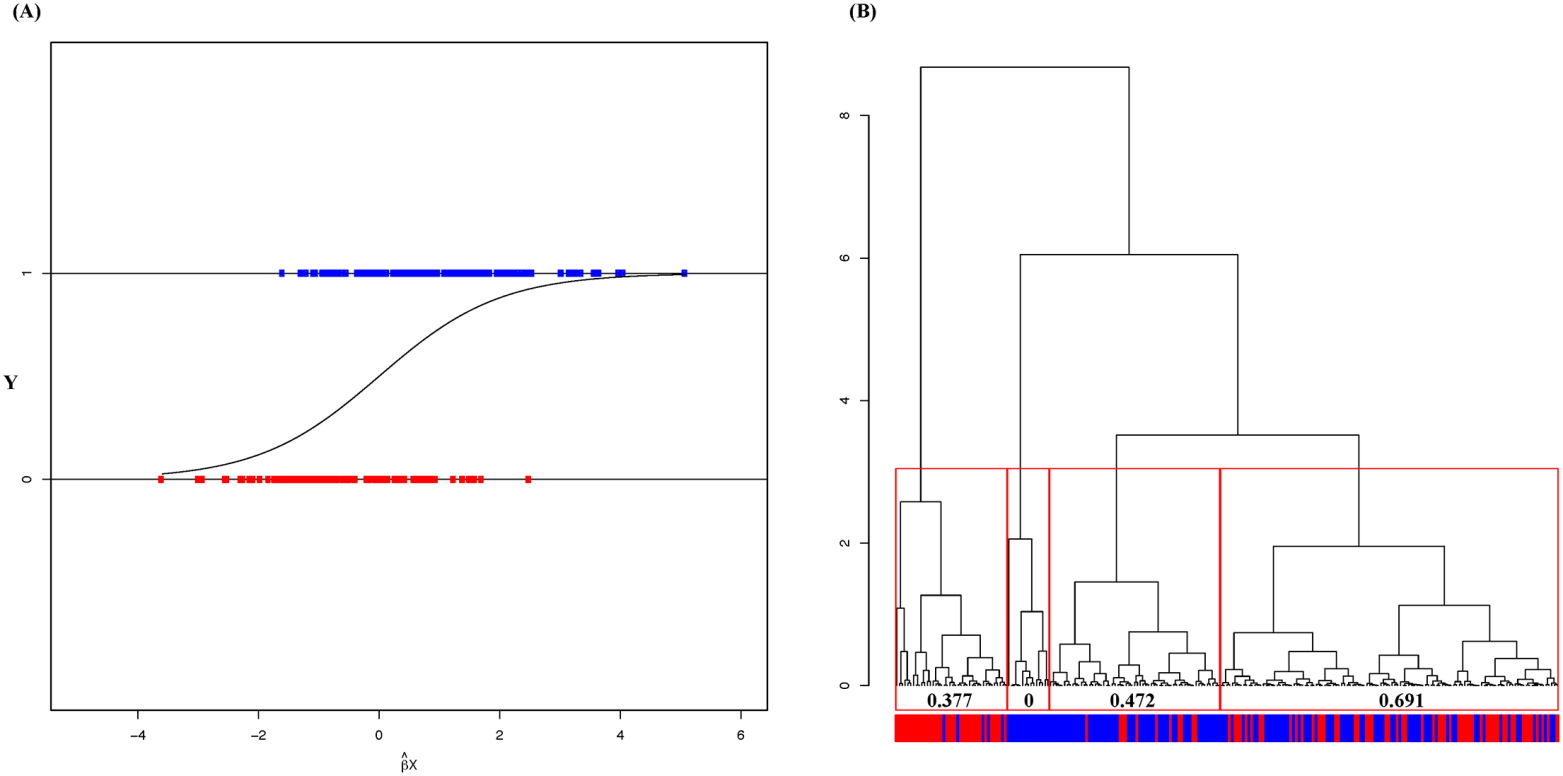
An expandable logistic regression setup and possibly heterogeneity. (A) Binary horizontal layout with respect to $\hat{\beta }X_{i}$ with MLE $\hat{\beta }$.; (B) Histogram of $\hat{\beta }X_{i}$ with calculated entropies for each cluster. A high degree of overlapping between the two horizontal layout in (A) indicates inefficiency of Logistic regression. In contrast, the heterogeneity within each gender categories in (B) gives rise to precise results in clusters of $\hat{\beta }X_{i}$ with low entropies.

A non-classical view of a Logistic regression model is expressed in Fig 1(A) with two horizontal half-lines being designated for the binary response categories: *Y* = 1 and *Y* = 0, while linear combinations of covariates *βX* are correspondingly marked on these two half-lines. With respect to this display, the optimal *β* is seen to achieve the least overlapping between the two ranges: *R*_*e*_ [1] = [min{*βX*_*i*_|*Y*_*i*_ = 1}, max{*βX*_*i*_|*Y*_*i*_ = 1}] and *R*_*e*_[0] = [min{*βX*_*i*′_|*Y*_*i*′_ = 0}, max{*βX*_*i*′_|*Y*_*i*′_ = 0}]. This non-classical view is indeed fundamental because its expanded version of display can accommodate the setting of response variable *Y* having more than two categories. With such a fundamental view, why the Logistic regression model is still not expandable? The answer essentially lies with the simple fact that even the straight forward overlapping evaluation among all induced ranges can’t afford a single smooth functional form of *β*.

Apart from being not able to accommodate a response variable beyond binary response variable, a Logistic regression also critically suffers from its linearity imposed constraint of homogeneity. It can’t accommodate heterogeneity such as shown in the Fig 1(B). The hierarchical clustering tree of ${\hat{\beta }}^{\left(MLE\right)}X$ at its 4-cluster tree-level is capable of revealing heterogeneous information contents. That is, instead of counting the overlapping *R*_*e*_[1] and *R*_*e*_[0], indeed we can extract more information by breaking the range *R*_*e*_[1]⋃*R*_*e*_[0] into pieces in a natural way. Informative patterns are observed upon these four clusters (from the left to the right) as follow:1) primarily dominant by Red color-coded subjects (*Y* = 0); 2) purely Blue color-coded subjects (*Y* = 1); 3) primarily dominant by Blue color-coded subjects (*Y* = 1); 4) a mixture of Red and Blue color-coded subjects. It is surprising that by allowing heterogeneity, the misclassification result of a Logistic regression with a given threshold can be very much improved and more precisely understood. This hierarchical clustering tree provides an extra advantage that there is no need to choose an ad hoc threshold to count for the false-positive (FP) and false-negative(FN).

However, the heterogeneity through the hierarchical clustering tree of ${\hat{\beta }}^{\left(MLE\right)}X$ provides only one limited aspect of intrinsic heterogeneity contained within the data because of being limited by one specific direction of covariate features pertaining to ${\hat{\beta }}^{\left(MLE\right)}$. Hence, it is realistic to expect that, if data’s whole intrinsic heterogeneity is properly computed and suitably represented and visualized, then the full information contents contained within data should be seen. Here such intrinsic heterogeneity is taken as system knowledge. In order to reveal such heterogeneity fully, as the ultimate goal of our data driven computations in this paper, we advocated unsupervised learning and computing paradigms, as would be briefly described below. It is worth emphasizing that the importance and essence of such paradigms is to make all computed results free from man-made constraints or distortions via invalid modeling assumptions. This important and essential point is particularly relevant to data analysis in the age of big data.

**Fig 2:**
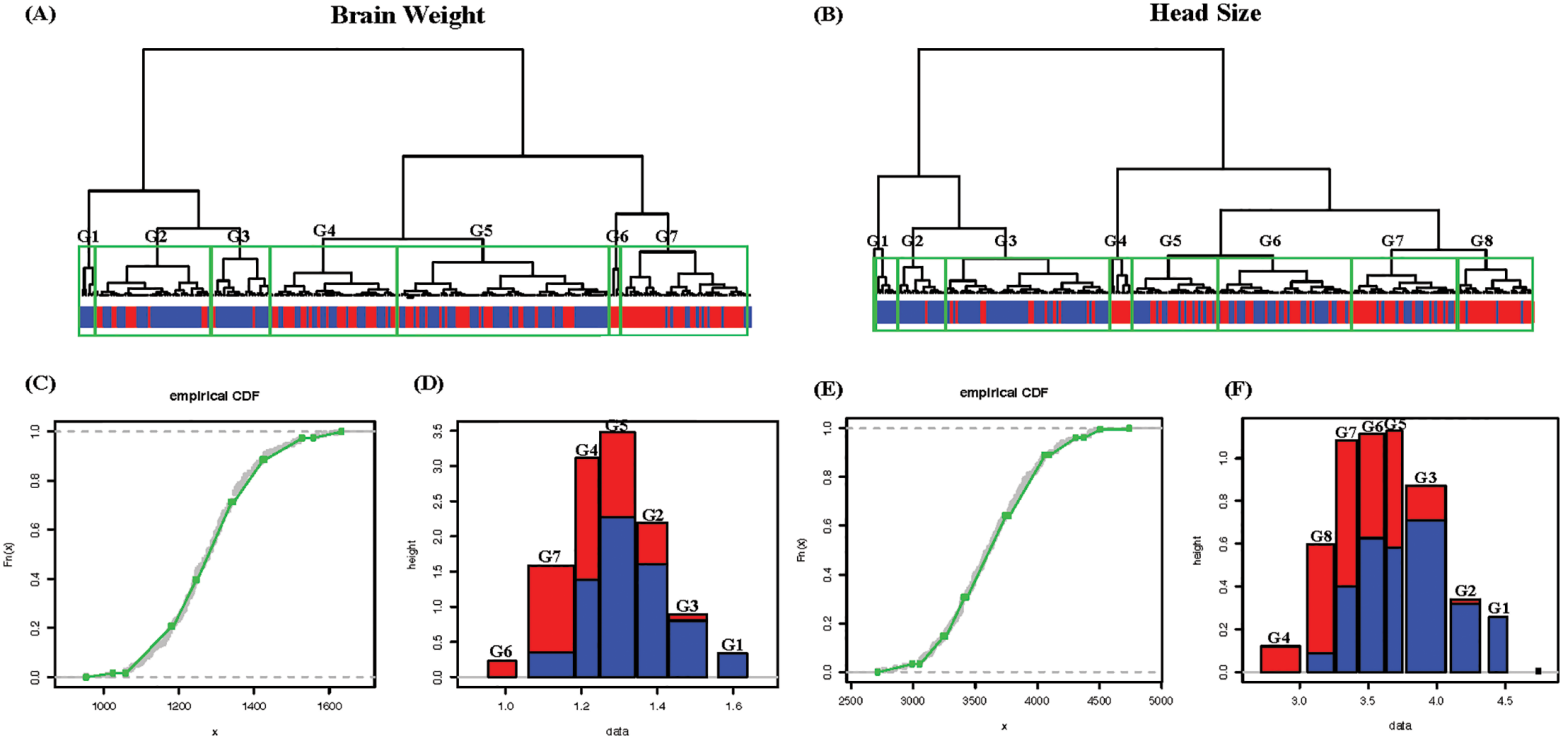
Two features’ hierarchical clustering trees and corresponding empirical distributions and possibly-gapped histograms. (A)Brain weight’s hierarchical clustering tree marked with 7 clusters; (B)Head size’s hierarchical clustering tree marked with 8 clusters; (C)The empirical distribution of head size superimposed with an 8-piece linear approximations showing with possibly-gaps; (D) The possibly-gapped histogram with 8 bins colored with gender proportions. (E)The empirical distribution of brain weight superimposed with a 7-piece linear approximations showing with possibly-gaps; (F) The possibly-gapped histogram with 7 bins colored with gender proportions. It is noted that the both histograms in (D) and (F) have two visible gaps separating the far-left and far-right bins. This is the strong evidence of dependency between these two features. The Red color code is for female and Blue for male.

**Possibly-gapped histogram based re-normalization** Let $M_{0}$ be an observed *n* × *m* data matrix with *n* subjects being arranged along the row-axis and *m* features along the column-axis. Each feature specific column has to undergo a digital re-normalization procedure based on a data-driven possibly-gapped histogram as illustrated in Fig 2, see detailed computations and algorithmic programs in [4]. Such a possibly-gapped histogram based re-normalization is designed to achieve three goals of making:1) all columns free from their idiosyncratic measurement scales; 2) all features’ ranges comparable; 3) digital coding naturally reflecting the 1D data-structural geometry. In summary, a feature’s possibly-gapped histogram has to effectively approximate its empirical distribution function, which may have horizontal gaps. That is, no continuity assumption is implicitly imposed here. By doing such a re-normalization, all features involved could possibly be able to contribute nearly equally to the similarity or distance measurements of all feature-pairs as well as all subject-pairs.

However, when the *m* features are mixed in data-types: continuous, discrete and categorical, as would be seen in the Heart data below, the re-normalization becomes a rather tricky issue to be resolved in order to achieve a large degree of uniformity. Here we suggest a guideline: **two features with relatively low mutual conditional-entropy should be similarly digitally coded**. Denote the *n* × *m* re-normalized data matrix be $M_{®}$.

**Synergistic-vs-antagonistic feature grouping via Data Cloud Geometry (DCG) algorithm.** Upon this *n* × *m* re-normalized data matrix $M_{®}$, we can compute a *m* × *m* mutual conditional-entropy matrix, say Ξ, for all feature-pairs (*Y*, *X*), which is generic bivariate digital coding, i.e. two separate columns of $M_{®}$, as follows:

$$\begin{array}{c} 2E\left[Y⟺X\right]=\frac{E[E_{x}^{\left(0\right)}\left(Y\to X\right)]}{E^{\left(0\right)}\left(Y\right)}+\frac{E[E_{y}^{\left(0\right)}\left(X\to Y\right)]}{E^{\left(0\right)}\left(X\right)}=E\left[Y\to X\right]+E\left[X\to Y\right], \end{array}$$

with *x* ∈ {*a*, *b*, *c*, ‥} and *y* ∈ {*A*, *B*, *C*, …} according to notations in S2 Box. It is noted that the conditional entropy $E[E_{a}^{\left(0\right)}\left(Y\to X\right)]$, also conventionally denoted by *H*(*Y*|*X*), is evaluated with respect to the discrete distribution of *X*, while the entropy *E*^(0)^(*Y*), also conventionally denoted by *H*(*Y*), is calculated with respect to the discrete distribution of *Y*.

Then the *m* × *m* mutual conditional-entropy matrix Ξ can be taken as a distance matrix for feature-grouping computations. The Data-Cloud-Geometry (DCG) computing algorithm employed here aims at building an Ultrametric clustering tree, say T[Ξ]. The key concept underlying DCG algorithm is to discover multiple essential scales, to which clustering relational patterns are evident. The DCG computing is heuristically analogous to the microscope operating in the process of finding out multiple scales of cell-structures: we need to tune to one right resolution in order to see one particular scale of structure, then we tune to another right resolution for another scale of structure. As such natural clustering compositions must be scale-dependent and need to be discovered. When they are synthesized with respect to decreasing identified scales, an Ultrametric clustering tree is built with one scale corresponding to one tree level. A version of DCG algorithm is given below for convenience of readers, see detailed algorithmic computing in [7] [8].

**DCG algorithm:** We begin with taking mutual the conditional entropy matrix Ξ = [*d*_*ij*_] as a distance matrix. In general a distance matrix is derived through a distance measure, which is typically an empirical choice of system scientists.

Step-1 With respect to a temperature (or scale) *T*, which is typically chosen with respect to the histogram of all entries of Ξ = [*d*_*ij*_], a similarity matrix is generated as $S^{T}\left(D\right)=\left[s_{ij}^{T}\right]$ with $s_{ij}^{T}=e^{\frac{-d_{ij}}{T}}$.
Step-2 Then each row of *S*^*T*^(Ξ) is normalized by its row sum. So *S*^*T*^(Ξ) becomes a transition probability matrix *P*^*T*^(Ξ).
Step-3 *P*^*T*^(Ξ) gives rise to a regulated Markov random walk, which starts randomly from a leaf-node, and then removes a leaf-node, when its number of visits by this Markov random walk has gone beyond a threshold. When a leaf-node is removed, the transition matrix *P*^*T*^(Ξ) is regenerated by deleting the corresponding row and column. This step is designed to keep the Markov random walk from being trapped in a local region of the data cloud.
Step-4 A trajectory of a regulated random walk will give rise to a leaf-node-removal recurrence time series, which is equipped with several spikes, which indicating the random walk has just enters a new, unexplored local region. Therefore, all leaf-nodes removed between two successive spikes are taken as being in the same cluster with respect to the temperature *T*.
Step-5 So a trajectory will give rise to a binary matrix: its (*i*, *j*) entry is coded 1 if the *i*–th and *j*-th leaf-nodes are in the same cluster. An ensemble of such trajectories will give rise to an ensemble of such cluster-sharing matrices, which is then summarized into a matrix of cluster sharing probability, denoted as *En*[*P*^*T*^(Ξ)]. (It is noted that this $\tilde{1}{\tilde{1}}^{T}-En\left[P^{T}\left(\Xi \right)\right]$ is nearly an Ultrametric, which satisfies the super-triangular inequality: *d*(*x*, *y*) ≤ max *d*(*x*, *z*), *d*(*y*, *z*).)
Step-6 The number of significantly non-zero eigenvalues of *En*[*P*^*T*^(Ξ)] is taken as the number of clusters, say *N*(*T*), being present in the temperature scale *T*, while the explicit clustering composition can be extracted by applying HC algorithm, or other clustering algorithm based on $\tilde{1}{\tilde{1}}^{T}-En\left[P^{T}\left(\Xi \right)\right]$ as a distance matrix. Denote the resultant clustering composition as $C_{M}\left(T\right)$, which contains the memberships of the *N*(*T*) clusters.
Step-7 Plot *N*(*T*) against *T* (on horizontal axis). We choose a temperature *T*_*i*_ from each leveling-off or constant segment of this plot. (Typically we choose the middle point.) Denote this set of selected temperatures as {*T*_1_, …, *T*_*K*_} and their corresponding clustering compositions $\left\{C_{M}\left(T_{1}\right),...,C_{M}\left(T_{K}\right)\right\}$. This set of clustering compositions are synthesized into a Ultrametric clustering tree. This tree is called Data Could Geometry (DCG) tree, say T[Ξ].

By superimposing this Ultrametric tree on row and column axes of Ξ, its framed matrix lattice will naturally show multiscale block-patterns, as illustrated in panel(A) of S3 Box with four color-coded synergistic feature groups. The blocks on diagonal of various sizes are blocks consisting of relative small mutual conditional-entropies, so they are synergistic feature-groups with various degrees. In contrast, off-diagonal blocks having large mutual conditional-entropies indicate antagonistic relations between feature-groups. In summary, the chief merit of mapping out synergistic feature-groups against antagonistic ones upon a mutual conditional entropy matrix is to discover which features will group with which features to constitute potential complex nonlinear dependency structures, and at the same time to figure out which feature-groups are potentially related upon a higher level, and which are antagonistic.

Here it is worthy clarifying and reiterating the conceptual nature of two features or feature-groups being “synergistic vs antagonistic”. These two contrasting concepts simply refers to increasing or decreasing “potentials” of pattern formation of dependency between the two features or feature-groups. As “dependency” is naturally revealed through “categorical” correspondence, which is typically visible when two features or feature-groups are put together side-by-side under an unsupervised learning setting. The strong categorical correspondence is exactly the phenomenon conveyed by being synergistic, and is precisely captured and measured by having low mutual conditional entropy. Specifically they are “categorically” corresponding to each other in a way that, by knowing a category of one feature upon a subject, we can predict very well about which category of the other feature upon the same subject will belong to.

In contrast, this good predictability disappears when two features are indeed antagonistic. That is, two antagonistic features are lacking “categorical” correspondence, so not only patterns of dependency can hardly emerge when they are put together side-by-side, but also they in fact tend to destroyed patterns of individual feature or feature-groups. This potential of destroying patterns is what “antagonistic” is referring to.

Furthermore we remark that the synergistic and antagonistic correspondences can be highly non-linear. However, if such correspondences are in fact linear, then the synergistic correspondence is equivalent to correlations going either highly positive or highly negative, while the antagonistic correspondence is equivalent to nearly zero correlation.

**Data mechanics on matrix data** Features sharing a synergistic feature-group are highly dependent because they potentially share the same mechanism within the study system. Such dependency will allow unsupervised learning algorithms to more effectively reveal fine scale interacting relational patterns between subject-clusters and feature-clusters. That is, the row-axis of $M_{®}$ should be partitioned according to the hierarchy of T[Ξ], so that different involving mechanisms are discovered and visualized, as seen in panel(A) of S3 Box.

The unsupervised learning algorithm employed here is called Data Mechanics (DM), see Fushing and Chen (2014) and Fushing et al. (2015). Data Mechanics is an iterative algorithm that build one DCG-based Ultrametric tree on row-axis and one on column-axis alternatingly. In each iteration, **distance metrics are updated with respect to the tree structure obtained from the previous step on the other axis**. Denote the final two Ultrametric trees $T{\left[M_{®}\right]}_{R}$ and $T{\left[M_{®}\right]}_{C}$ on row- and column-axes, respectively. The overall goal of DM computing is to permute rows and columns such that multiscale blocks framed by the two marginal trees $T{\left[M_{®}\right]}_{R}$ and $T{\left[M_{®}\right]}_{C}$ are as uniform as possible. A version of DM algorithm is given below for convenience of readers, see detailed algorithmic computing in [5] [6].

**Data Mechanics:**

Step-1 We adopt the Euclidean distance measure on all *m* rows of $M_{®}$, and construct a distance matrix $D_{R}^{\left(0\right)}=\left[d_{ij}^{\left(R0\right)}\right]$. We then apply DCG algorithm to build an initial version of Ultrametric tree, say $T{\left[M_{®}\right]}_{R}^{\left(0\right)}$ on the row axis.
Step-2 Select a tree level on $T{\left[M_{®}\right]}_{R}^{\left(0\right)}$. Extract the corresponding clustering composition $C(T^{*}|T{\left[M_{®}\right]}_{R}^{\left(0\right)})$ with N* clusters. Consider each column being extended with N* extra dimensions of average among member-rows of clusters of $C(T^{*}|T{\left[M_{®}\right]}_{R}^{\left(0\right)})$. Define a distance matrix $D_{C}^{\left(1\right)}=\left[d_{i^{\prime }j^{\prime }}^{\left(C1\right)}\right]$ among the *n* column with $d_{i^{\prime }j^{\prime }}^{\left(C1\right)}$ being calculated as the *m* + N* dimensional Euclidean distance.
Step-3 Based on distance matrix $D_{C}^{\left(1\right)}$, a DCG tree $T{\left[M_{®}\right]}_{C}^{\left(1\right)}$ is calculated on column axis.
Step-4 Based on one selected level of the tree $T{\left[M_{®}\right]}_{C}^{\left(1\right)}$, an adopted distance measure, say $d_{ij}^{\left(R1\right)}$, on row vectors are devised as in Step 2, and a new distance matrix $D_{R}^{\left(1\right)}=\left[d_{ij}^{\left(R1\right)}\right]$ is also computed. We then apply DCG algorithm to build a new Ultrametric tree, say $T{\left[M_{®}\right]}_{R}^{\left(1\right)}$ on the row axis.
Step-5 Repeat the Step-2 to Step-4 for two or three times, or until both trees $T{\left[M_{®}\right]}_{R}^{\left(k\right)}$ and $T{\left[M_{®}\right]}_{C}^{\left(k\right)}$ are stable.
Step-6 Let the final two marginal trees are denoted as $T{\left[M_{®}\right]}_{R}$ and $T{\left[M_{®}\right]}_{C}$. The final result of Data Mechanics computations is a heatmap described by superimposing the two marginal Ultrametric trees $T{\left[M_{®}\right]}_{R}$ and $T{\left[M_{®}\right]}_{C}$ of the row and column axes of $M_{®}$. These two tree jointly frame the multiscale block patterns on the lattice of $M_{®}$, which is termed a heatmap of coupling geometry. Ideally all blocks on the finest scale embed with uniformity.

The uniformity within each of the finest scale block collectively forms the stochastic structures contained within $M_{®}$, while the multiscale blocks framed by two marginal trees $T{\left[M_{®}\right]}_{R}$ and $T{\left[M_{®}\right]}_{C}$ constitutes the deterministic structures contained within $M_{®}$. These two coupled structural components are taken to be the information content and termed coupling geometry of $M_{®}$. The DM and its coupling geometry are illustrated through panels (B) and (C) of S3 Box with three heatmaps corresponding to three iterations, respectively. The heatmap from 1st iteration of DM is apparently very much improved by that of 2nd and 3rd iterations. The later two are exactly the same. This fact indicates that the number of iterations needed is in general small.

**Organization of knowledge via information flows** Now consider a *n* × *m*_*Re*_ response data matrix, $M_{0}^{\left(Re\right)}$, and one *n* × *m*_*Co*_ covariate data matrix $M_{0}^{\left(Co\right)}$. The ultimate computing goals are to identify all essential system mechanisms involving both response and covariate sides, and then discover all related knowledge through directed associative patterns that link a response mechanism to a serial covariate mechanisms. The computations for achieving such goals are carried out in the following steps.

**[Algorithmic steps for discovering and confirming information flows:]**

1. **[Re-normalizing all features]:** Matrices $M_{0}^{\left(Re\right)}$ and $M_{0}^{\left(Co\right)}$ will undergo their column-by-column re-normalization, as described in the above paragraph. The resultant digital-coding matrices are denoted as $M_{®}^{\left(Re\right)}$ and $M_{®}^{\left(Co\right)}$, respectively.
2. **[Re-grouping synergistic features]:** Upon $M_{®}^{\left(Re\right)}$, its *m*_*Re*_ × *m*_*Re*_ mutual conditional-entropy matrix Ξ_*Re*_ is computed, so is *m*_*Co*_ × *m*_*Co*_ mutual conditional-entropy matrix Ξ_*Co*_ based on $M_{®}^{\left(Co\right)}$. Then essential mechanisms on response and covariate sides are identified through DCG-based Ultrametric clustering trees $T[\Xi_{Re}]$ and $T[\Xi_{Co}]$, respectively. Hence, synergistic feature-groups on response and covariate sides are collected respectively.
3. **[Discovering block-patterns via Data Mechanics]:** Each data submatrix of $M_{®,i}$ corresponding to each synergistic feature-group would undergo Data Mechanics computations, and its row-marginal tree $T{\left[M_{®,i}\right]}_{R}$ is collected.
4. **[Exploring information flows via Categorical-pattern matching]:** Each row-marginal tree $T{\left[M_{®,i}^{\left(Re\right)}\right]}_{R}$ on the response side will be paired with one row-marginal tree $T{\left[M_{®,j}^{\left(Co\right)}\right]}_{R}$ on the covariate side, and compute the directed response-to-covariate conditional entropy as *E*[*Y* → *X*]. A low value of such directed conditional entropy implies that there exist strong associative patterns. Specifically a strong associative pattern is identified as a cluster or branch of $T{\left[M_{®,j}^{\left(Co\right)}\right]}_{R}$ being nearly exclusively belonging to one particular cluster or branch of $T{\left[M_{®,i}^{\left(Re\right)}\right]}_{R}$. Such a graphic display of associative pattern has the capability of fostering understanding, so is taken as a locus of system knowledge.
5. **[Information flows]:** Organize all associative patterns with respect to a series of coupling geometries via a series of heatmaps. This graphic display is called Information flow, which is taken as a representation of knowledge from one response mechanism to a series of covariate mechanisms.

**Confirming an information flow and calculating its error rate.** Due to the exploratory nature of an organization of directed associative patterns, a result of categorical-pattern matching, needs a formal confirmation, and then its error rate has to be evaluated. All these computations are scale-dependent, but rather simple and elementary. Here the scale-dependence is referring to a clustering composition of subjects pertaining to a chosen tree level of the row-marginal tree $T{\left[M_{®,i}^{\left(Re\right)}\right]}_{R}$ coupled with a clustering composition of subjects pertaining to a chosen tree level of row-marginal tree $T{\left[M_{®,j}^{\left(Co\right)}\right]}_{R}$.

Given a pair of clustering compositions respectively coming from response and covariate sides, observed directed conditional entropies are evaluated on each individual cluster of the clustering composition on the covariate side, as illustrated in S2 Box. To confirm any pattern contained in an information flow, we apply the simple random sampling without replacement (with respect to the proportions of subjects in the clustering composition on the response side) to calculate a simulated entropy distribution and accordingly the P-values with respect each observed entropy.

For an error rate of an information flow with only one covariate synergistic feature-group, either majority rule or randomized rule can be applied within each cluster on the covariate side to calculate individual cluster’s error rates, and then a weighted overall version is calculated with respect to sizes proportions of the covariate clustering composition. The reason underlying this simplicity is given as follows. Randomly select one subject and remove its cluster membership on the response side. The key is that based on the semi-unsupervised learning paradigm, this subject’s row covariate vector needs to participate in the construction of row-marginal tree $T{\left[M_{®,j}^{\left(Co\right)}\right]}_{R}$. Thus, this row-marginal tree $T{\left[M_{®,j}^{\left(Co\right)}\right]}_{R}$ is invariant with respect to any random choice of subject. That is, the selected subject keeps its original position in the clustering composition on the covariate side. Therefore, by repeating this random selection for a large number of times, the majority rule or any randomized rule will eventually give the expected error rates within each cluster and its overall one equal to their observed ones.

For an error rate of an information flow on serial covariate synergistic feature-groups, the overall error rate is calculated in a weighted fashion. The weighting should be inversely proportional to their individual conditional-entropies. Such simplicity is one significant advantage of adopting an unsupervised learning paradigm. That is, here there is no need to perform the cross-validation as needed in supervised learning paradigms.

## Results

In this section we analyze five simple system data sets from UCI Machine Learning Repository

(https://archive.ics.uci.edu/ml/datasets.html). Each data set is chosen for idiosyncratic reasons and characters: 1) the 1st data set with 1D binary response feature is to show why an information flow is more advantageous over Logistic regression model; 2) the 2nd data set with 1D continuous response feature is to recognize the fact that a data set can only sustain limited, not full spectrum, of resolutions of information content as implied by a linear regression model; 3) the 3rd and 4th data sets deal with multiple response features with distinct data types; 4) the 5th data set consists of covariate features of all types: from continuous, discrete to categorical ones, in which all features need to be properly digitally coded.

Here all results of the five data sets presented via information flows are meant to advance our system knowledge with concise and vivid pictorial visualizations. Such an organization of associative patterns has the potential to take human and machine learning to the next technical level.

**Brain weight and head size data** The first data set from [13] consists of two covariate features: 1)brain weight (grams); 2) and head size (cubic cm), for 237 adults classified by two response features: binary gender and age groups: 1) Gender: 1 = Male, 2 = Female; 2) Age Range: 1 = 20 ∼ 46, 2 = 46^+^. Data can be found via links: http://users.stat.ufl.edu/~winner/data/brainhead.dat, and http://users.stat.ufl.edu/~winner/data/brainhead.txt.

An extended version of Logistic regression of gender on brain weight and head size is reported in Fig 1(A) with $\hat{\beta }X_{i}$ on the horizontal axis. Here $\hat{\beta }$ is the maximum likelihood estimates (MLE) based on Logistic regression model. The evident high degree overlapping between *R*_*e*_[1] and *R*_*e*_[0] confirms the inefficiency of Logistic regression on these data. The error rate is 28.3% with threshold at 0.5. This inefficiency due to the artificially imposed homogeneity structure is further contrasted with the four-cluster composition based on the HC tree of $\hat{\beta }X_{i}$. The three clusters (from the left to the right), except the 4th one, have rather low entropy.

The two possibly-gapped histograms of brain weight and head size are constructed and color-coded with gender-counts into each bin, as shown in Fig 2(A) and 2(B), respectively. Each histogram reveals obvious gaps on the two sides of extreme. The heterogeneity manifested through the color-coding and presence of gaps strongly indicates that any homogeneity based on a single distribution assumption is not valid, and more importantly, goes against the true nature of data. Hence, such evidence indicates that Logistic regression is not correct for this data set.

**Fig 3:**
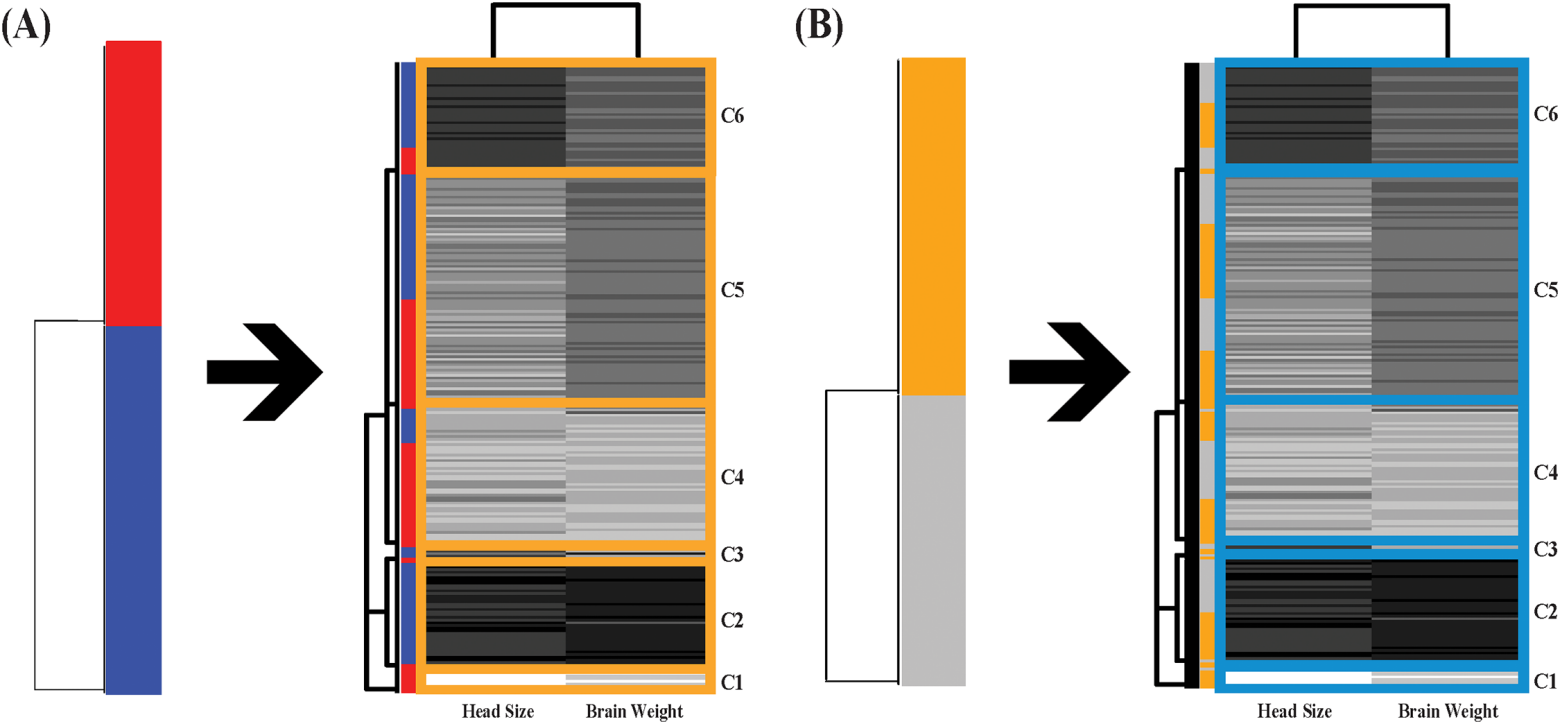
Information flows; (A)for binary-gender; (B)for binary-age. The information flow (A) shows rather evident associative patterns from the gender-tree with male- and female-specific clusters to the DCG-tree based on head size and brain weight with 6 clusters. Except one, all clusters have extremely or relative low entropies. This result shows the effectiveness of information flow over classic logistic regression. The information flows (A) and (B) share a cluster with extreme low values of the two features.

The mutual conditional-entropy *E*[*Y* ⇔ *X*] of the two response features: gender and age, is calculated as being nearly equal to 1. This large entropy value indicates that these two response features represent two separate mechanisms. Thus, two separate information flow are reported in Fig 3.

As shown in Fig 3(A), the gender’s information flow reveals very evident associative patterns: a) one extremely small brain weight and head size cluster(C1) is exclusively female; b) an extremely large brain weight and head size cluster(C2) is exclusively male; c) a cluster(C6) of large brain weight and large head size is dominant by male; d) a cluster(C4) of small brain weight and small head size is dominant by female; e) a cluster(C5) median brain weight and head size is mixed. Here we demonstrate that an information flow based on patterned dependency among covariate features can reveal the full spectrum of heterogeneity.

As shown in Fig 3(B), no signs of heterogeneity are seen in the age’s information flow, except the cluster(C1) of extremely smallest brain weight and head size. It is clear to see that this information flows can easily adapt to the setting of having more than two age-categories. That is, this information flow platform not only resolves the shortcomings of Logistic regression, but also provides a framework to substitute MNOVA and avoids the required unrealistic distribution assumption, its limitations and ambiguous interpretations altogether.

One of the original objectives of the investigation as reported in Gladstone (1905) was to obtain a series of reconstruction formulas to predict brain weight given measurement of head size. It is clear that, based on associative patterns of the three features via in the information flow shown in Fig 3(A), such a prediction would have heterogeneous degrees of precision by taking subject’s gender and cluster membership of head size into consideration.

As another demonstration of how dependency structures work, we classify the association between gender and head size into four groups (ranking from all female and extremely small head size to nearly all male and extreme large head size):1) G4; 2) G8; 3) G5-G7; and 4) G1-G3, as shown in panel (F) of Fig 2. Throughout these four groups, the information flow Fig 3(A) demonstrates that females’ categorical predictions of brain weight are all correct without ambiguity, while categorical predictions of brain weights for males in the 4th group (G1-G3), who have extremely large head size, can be either extremely heavy or just median heavy. Except such ambiguity on male’s prediction, other categorical predictions are rather precise.

**Fig 4:**
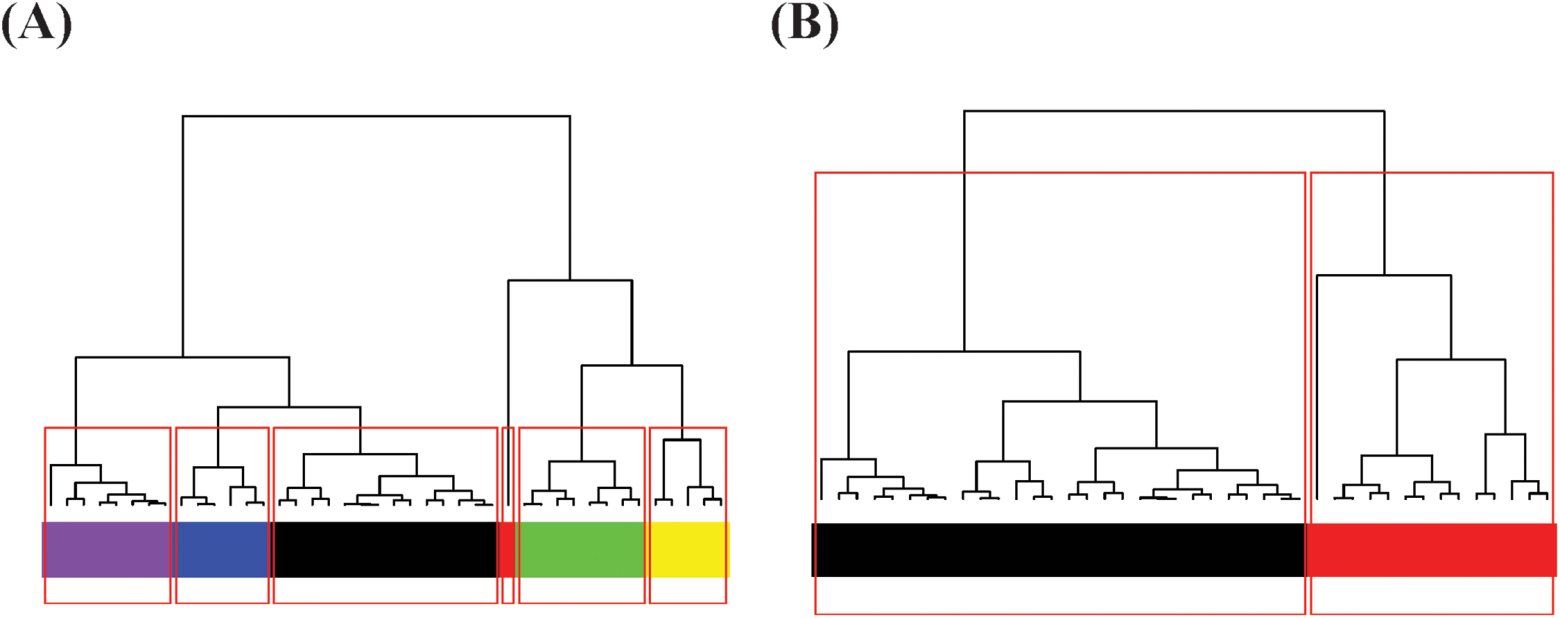
Response hierarchical clustering trees: (A) the fine-scale with 6 color-coded clusters; (B)the coarse-scale 2 color-coded clusters. It is intuitive that the task of successfully differentiating among the 6 fine-scale clusters of (A) would need much more covariate information than the task of differentiating between the two coarse-scale clusters of (B).

**Electricity data** The data from [14] represents electricity Consumption of 42 provincial town in Great Britain in 1937-1938. In each town one single response feature was observed: Average total expenditure on electricity, while there were 12 covariate features measured ranging from Average number of consumers(V1), percentage of consumers with two-part tariffs in 1937-38(V2), Average income of consumers(V3), prices on domestic tariffs in 1933-34(V4), 1935-36(V5), 1937-38(V6), Marginal price of gas 1935-36(V7), 1937-1938(V8), Average holdings of heavy electric equipment bought (V10) and per two-part consumer consumption 1937-38(V10), 1935-36(V11) and 1933-34(V12). Here we report information flows according to two scales of clustering on the response feature: one fine-scale (with 5 clusters and one extreme outlier) and one coarse-scale (with two clusters) clustering compositions of the response feature, as shown in Fig 4(A) and 4(B) respectively. Data can be found via links: http://users.stat.ufl.edu/~winner/data/gbelec.dat, and http://users.stat.ufl.edu/~winner/data/gbelec.txt.

**Fig 5:**
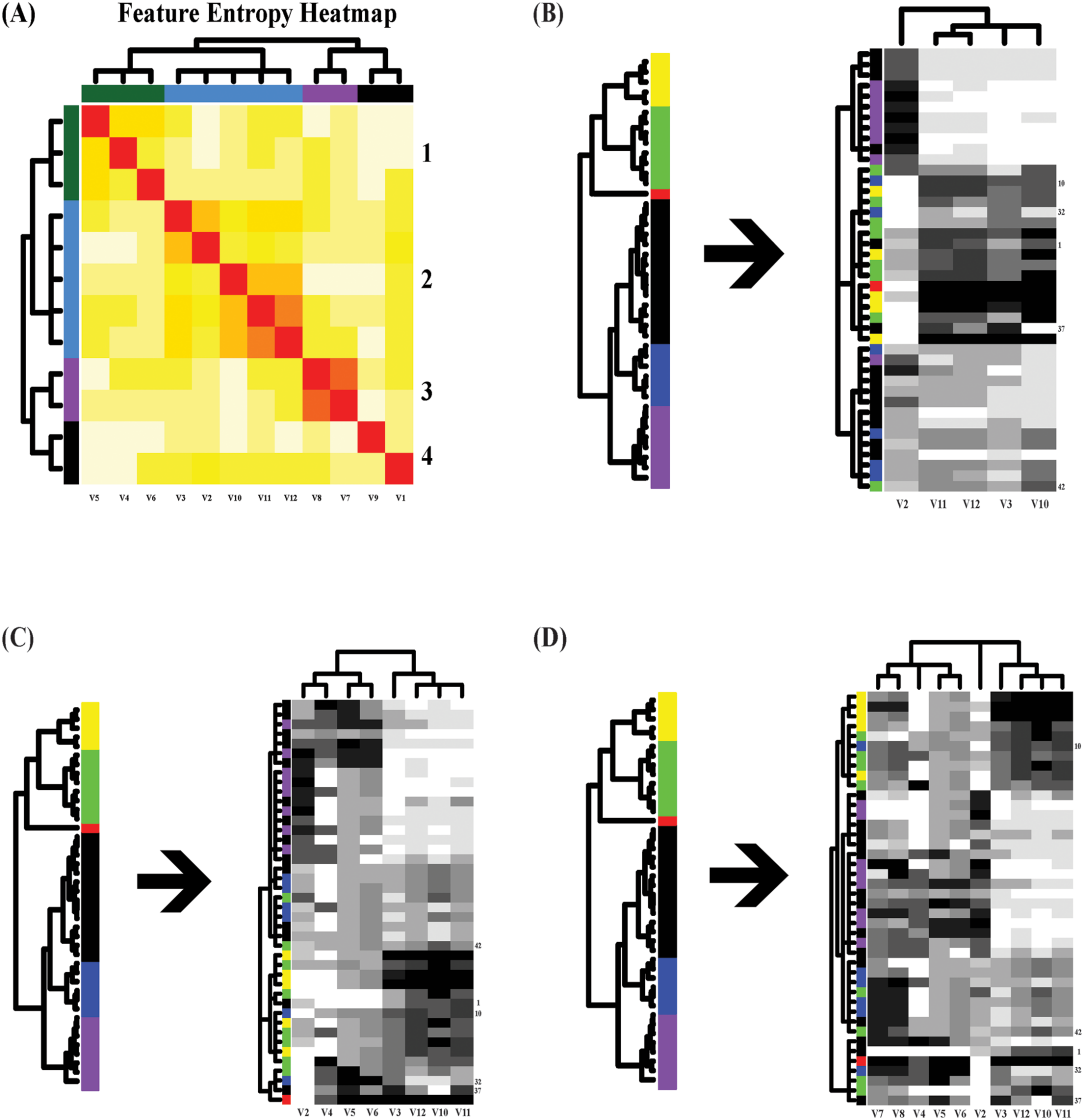
Information flows from response's fine-scale perspective. (A) Mutual conditional-entropy matrix superimposed with DCG tree with 4 synergistic feature-groups; The information flows from the response to (B) #2 synergistic feature-group; (C) #1 and #2; (D) #1 and #2 and #3 synergistic feature-groups. The misclassified subjects' ID numbers are attached to the right side of each heatmap.

The heatmap of mutual conditional-entropy of 12 covariate features, which is superimposed by a DCG tree, shows four synergistic feature groups in Fig 5(A). Three information flows from response’s fine-scale perspective are reported in three panels in Fig 5(B)-5(D). The information flow from the response to the synergistic feature-group#2 (V2, V3, V10-V12), as shown in Fig 5(B), demonstrates that each branch of the three-cluster level of DCG tree $T{\left[M_{®,2}^{\left(Co\right)}\right]}_{R}$ is coupled with rather clear dependency structures marked by uniform and evident block patterns in the heatmap.

Though each of these three cluster indeed consists of mixed color-coded memberships of the three response’s clusters, two out of three of them are significantly non-random. Their observed conditional-entropies are calculated (with P-values in parenthesis) as (0.65(0.0), 1.39(0.38), 1.05(0.004)) (from top to bottom on the 3-cluster level) in relative to the entropy on the response side calculated as 1.62. The p-values are evaluated through the simulation scheme of simple random sampling without replacement on the subject space with respect to the response’s 6 color-coding. Hence, we conclude that the presences of relative low entropy-values with extremely low p-values strongly indicate that response-to-covariate associative patterns are evident, but not exclusive.

Similar conclusions can be made for the other two information flows: 1) one union of #1 and #2 synergistic feature groups having 8 features, as shown in Fig 5(C); 2) and one union of #1, #2 and #3 synergistic feature groups having 10 features, as shown in Fig 5(D).

It is important, but not difficult to see that such non-exclusiveness in the middle covariate cluster of information flow in Fig 5(B), the bottom one in Fig 5(C) and the top one in Fig 5(D), is primarily due to the presence of three response categories with relative large values. This fact critically points out that this data set can’t sustain such a fine resolution on the response feature. Hence, we conclude that overall the fine scale structure with 6 clusters chosen for the response feature is supported only in part. In other words, this data set can’t afford such a fine scale separation on response features. How about the coarse-scale one?

**Fig 6:**
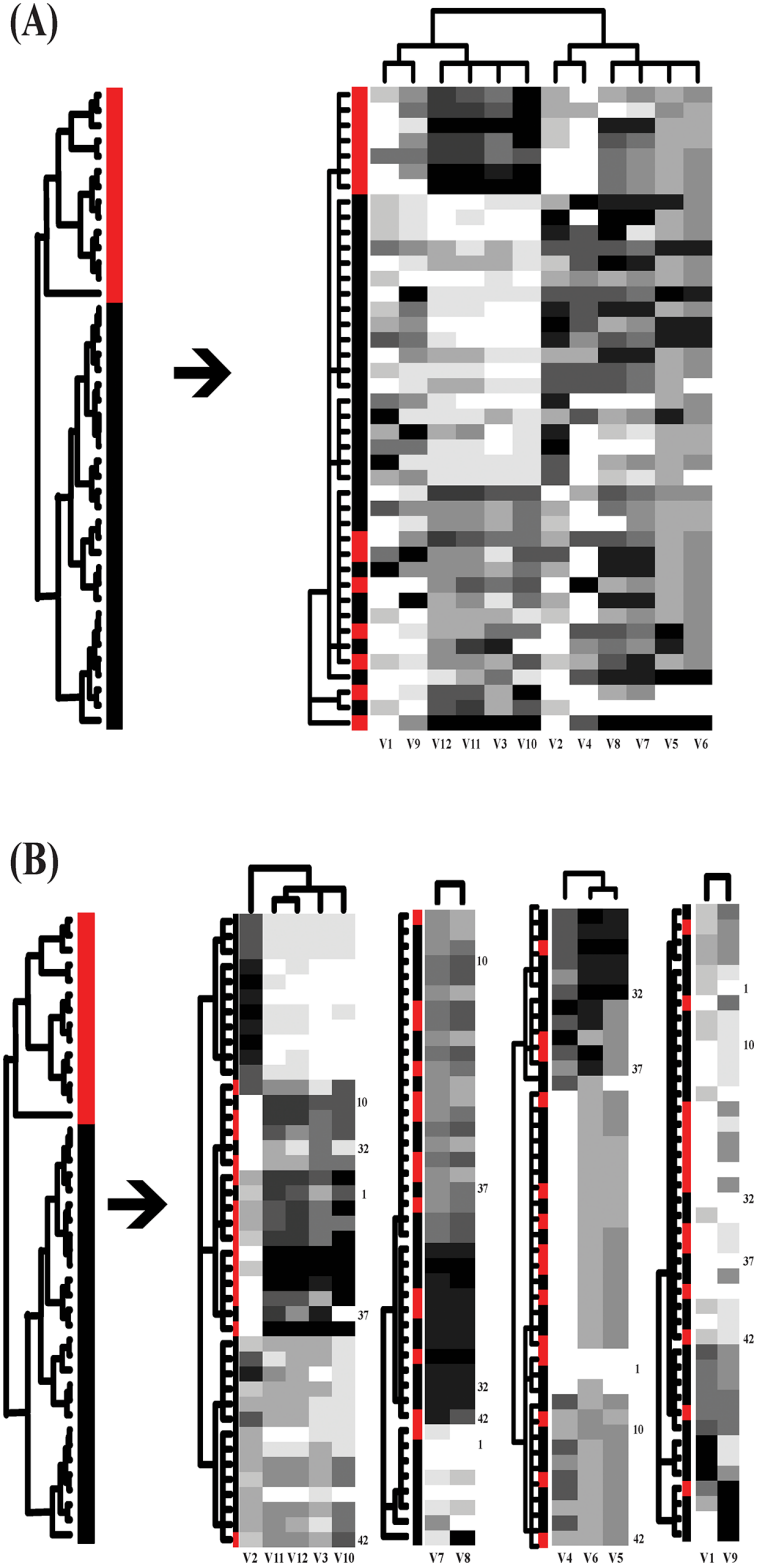
Information flows from response’s coarse-scale perspective. The information flows from the response to (A) #1&#2&#3&#4 synergistic feature-groups; (B) serial #2, #3, #1 and then #4 synergistic feature-groups.

Two information flows from response’s coarse-scale perspective are reported in Fig 6. Through the DCG tree $T{\left[M_{®}^{\left(Co\right)}\right]}_{R}$ (including all 12 features) superimposed upon a block-patterned covariate matrix, the information flow, as shown in Fig 6(A), reveals four major clusters are coupled with clear block patterns. Three of them have zero conditional-entropies by having exclusive memberships belonging to one of the two response clusters. However, the fourth one is a mixed.

In contrast the second information flow, as shown in Fig 6(B), the DCG tree $T{\left[M_{®,2}^{\left(Co\right)}\right]}_{R}$ based on #2 synergistic feature group pertaining to the first of the serial heatmaps on the right reveals a tree level with three clusters: 1) two exclusively contains members from the cluster of small-value cluster (in black) of response; 2) one is nearly exclusively dominated by members of the cluster of large-responses. That is, the exclusiveness of the linkage between response on coarse scale and covariate on three cluster scale is established.

Correspondingly two aspects of system understandings are derived as follows. The first aspect is that the small-value cluster of response contains heterogeneity caused by two block-patterns of covariate features: a) extremely large *V*2-value and extremely small (*V*3, *V*10 − *V*12)-values; b) median *V*2-value and median (*V*3, *V*10 − *V*12)-values. The second aspect is that the large-value cluster of response is attributed to extremely small *V*2-value and extremely large (*V*3, *V*10 − *V*12)-values. These two aspects spell out the first important part of associative patterns based on the clear dependency structures of synergistic covariate feature-Group #2. More associative patterns are available along the 2nd through 4th heatmaps of this information flow. These associative patterns can be used to further correct, or at least update and improve the misclassifications made in the 1st heatmap as follows.

Predictions are made and conformed via “majority rule” within each cluster identified across different heatmaps on the right. As illustrated in Fig 6(B), as if those number-marked subjects were missing their response feature measurements, then each heatmap gives rise to a set of predicted values. A final decision for each individual would be reached by simply conducting weighted averaging of the four predicted values with weights inversely proportional to the four corresponding conditional-entropy values. This is an error-correcting mechanism provided by using information flow with serial DM-computed heatmaps.

At the end of this example, it is strongly emphasized that the information flow in Fig 6(B) is much more proper and informative than the one in Fig 6(A) because the four synergistic covariate feature groups are somehow antagonistic to each other. Therefore, a platform for them to show their idiosyncratic dependency is needed. The information flow is designed to provide such a platform.

The original goal of this example according to [14] was two-fold: analyzing electricity demand and investigating monthly fluctuations. The hope was to incorporate results from these two parts to achieve a comprehensive study of the various features on electricity consumption. Yet this goal could not be realized in the original study due to insufficient information from the data as stated by the author. However, on the coarse scale of the response feature here, this goal can be achieved via our information flows. They indeed provide very comprehensive system understanding on the electricity consumption during the two year period from the 42 provincial towns in Britain.

**Patterns of Bird Species in Andes Mountains** In Ecology during 1970s, biogeographers assumed that continental biota found on high mountain tops are as isolated from one another as true islands. In order to test whether high mountain biota have insular distribution patterns, data of bird species was collected among “island” of mountain tops in 15 regions of the páramo vegetation in the Andes of Venezuela, Colombia and northern Ecuador [15]. Data can be found via links: http://users.stat.ufl.edu/~winner/data/brainhead.dat, and http://users.stat.ufl.edu/~winner/data/brainhead.txt.

There are 3 response features: Total Number of Species(V2), Number of species of South American origin(V3) and Number of endemic taxa(V4), and 7 covariate features: Area(V6), Base altitude(V7), Elevation(V8), Distance from Paramo(V9), Distance to nearest island of vegetation(V10), Distance to nearest island in south(V11) and Distance to nearest large island(V12), see details in [15].

**Fig 7:**
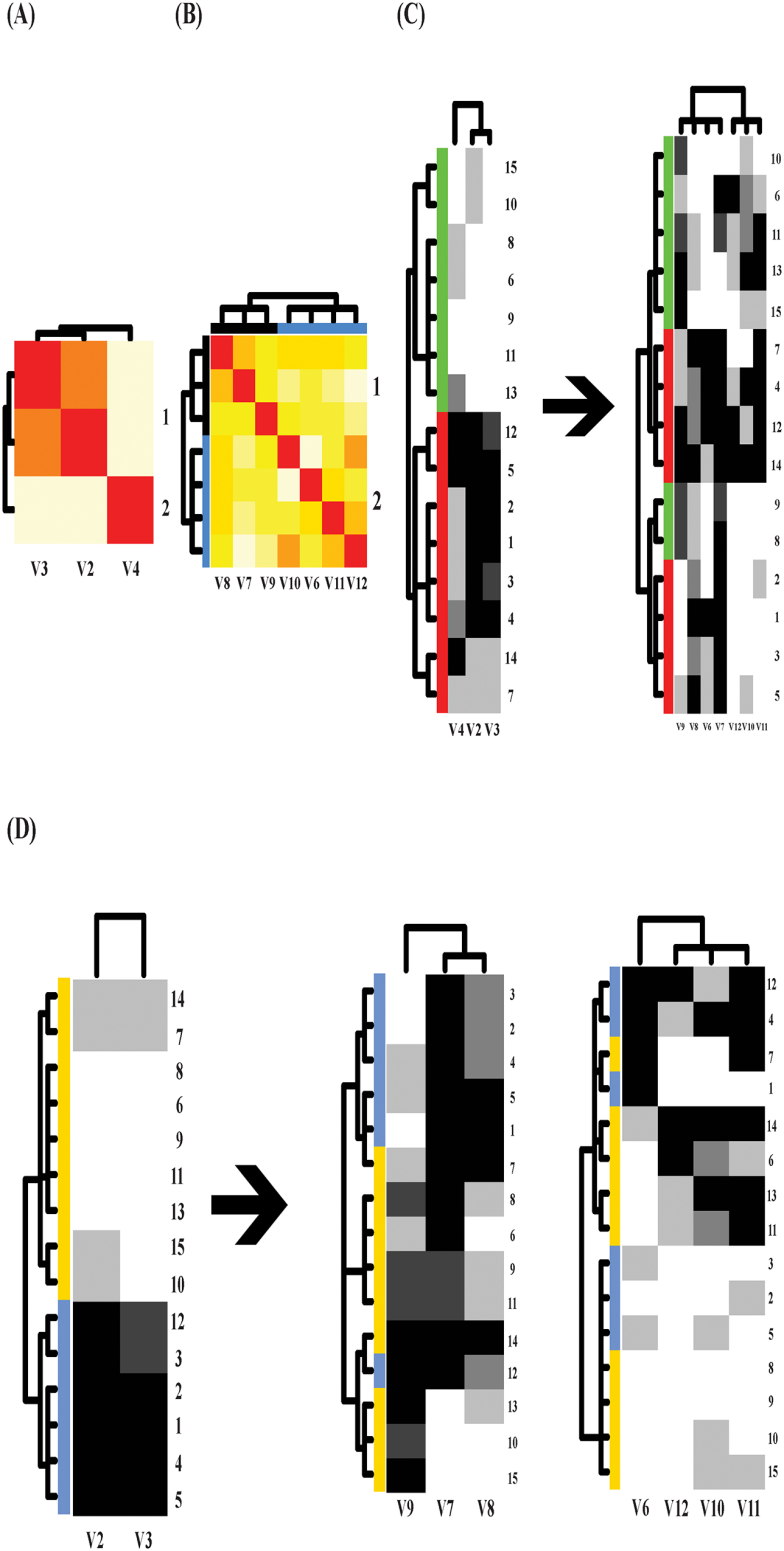
Mutual conditional entropy matrices and two information flows on bird data. (A) Mutual conditional entropy matrix of 3 response features divided into two synergistic groups; (B) 7 × 7 mutual conditional entropy matrix of covariate features with two color-coded synergistic groups; (C) Information flow from the response heatmap to the covariate heatmap showing heterogeneity; (D) Information flow from two response features v2 and V3 to two covariate heatmaps pertaining to the two synergistic groups.

Two mutual conditional-entropy matrices for the response and covariate features are separately computed, as shown in Fig 7(A) and 7(B). The response’s heatmap on left hand side of Fig 7(C) from DM clearly shows two patterned blocks that indicates strong joint dependency: largeness-vs-smallness, among response features. In contrast, the covariate’s heatmap on the right hand side of Fig 7(C) also clearly shows the joint dependency of 7-dim covariates in two scales: 1) two patterned blocks; 2) each block is intricately divided into two sub-blocks.

The first information flow linking the structural dependency on both sides, as shown in Fig 7(C), reveals a perfect linkage of heterogeneity from the response’s two blocks to the covariate’s 4 sub-blocks. This resulted perfect linkage of heterogeneity is surprising in the sense that the largeness-vs-smallness of response features is determined by rather intricate differences between sub-block belonging to each of the two block of covariate features. The second information flow in Fig 7(D) from 2-dim responses (V2 and V3) to a couple of 3-dim and 4-dim covariates also reveals the same kind of heterogeneity as clear as the first one.

Such splitting heterogeneity seen in the information flows conclude that the clearly bifurcated linkages from the response features to the covariate features strongly indicate that the high order dependency among covariate features is the driving forces underlying this biogeographic system, on one hand. On the other hand, it interestingly undermines the linear regression reported in the original paper.

The original investigation in [15] employed stepwise linear regression of one response feature at a time and reported very well statistical modeling fitting. Here we like to point out the fact that such statistical results likely were caused by over-fitting. The a linear hyper-plan based on 7 covariate features can easily over-fit the small number (15) of data points.

This example very well demonstrates the essence and importance of computing joint dependency among response and covariate features in order to discover evident heterogeneity through an information flow as shown in the Fig 7(C). Thus, it is worth emphasizing that the associative patterns contained in this data are organized on the fine, not coarse, scale of blocks. Since heterogeneity hardly can be accommodated by homogeneous linearity as assumed in the regression model, the results of linear regression analysis become misleading and dubious.

**Fig 8:**
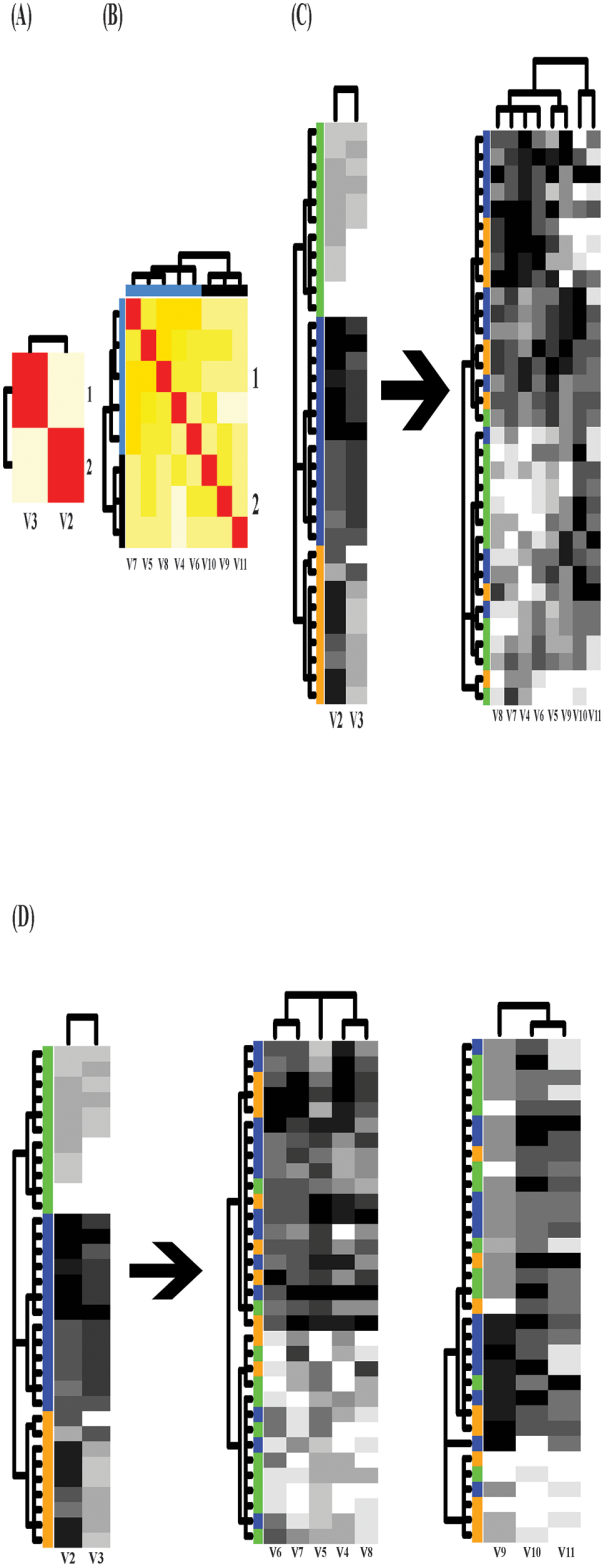
Information flow of height data. (A) and (B) for the mutual conditional-entropy matrices for response and covariate features; (C) Information flow to all covariate features; (D) Information flow to #1 feature-group and then #2 feature-group.

**Height and Various Stature Measurements Data** The fourth data set from [16] is consisting of 33 female police-department applicants. Each applicant has her standing Height(V2) and sitting height(V3) measured as two response features, and upper arm length (V4), forearm (V5), hand (V6), upper leg (V7), lower leg (V8), foot (V9), forearm/upper arm (V10), lower leg/upper leg (V11) are measured as seven covariate features. Mutual conditional-entropies on response and covariate sides are computed and shown in Fig 8(A) and 8(B), respectively. Two synergistic covariate feature-groups are identified:*Group*#1 = {*V*4, *V*5, *V*6, *V*7, *V*8} and *Group*#2 = {*V*9, *V*10, *V*11}. Data can be found via links: http://users.stat.ufl.edu/~winner/data/police_height.dat, and http://users.stat.ufl.edu/~winner/data/police_height.txt.

The response’s heatmap resulted from DM, as shown on the left hand side of Fig 8(C), clearly reveals three clusters coupled with evident block patterns. Thus, it is not only reasonable, but necessary to take both two dimensional features as response simultaneously. One information flow from the 2-dim response features to #1&#2 covariate features groups is carried out and reported in Fig 8(C). Also two major and one small covariate clusters are also supported by clear block patterns. One major covariate cluster is dominated by one response cluster members (Green color-coded) with a few coming from the the other two response clusters. We perform simple random sampling without replacement similar to permutation test to conform this pattern formation. The observed entropy is relatively small 0.87 comparing with 1.09 the overall entropy from response with the p-value 0.01. The other major covariate cluster primarily has mixed memberships of two response clusters (blue and orange color-coded). The observed entropy is 0.84 with its p-value 0.008.

The second information flow from the 2-dim response features to a serial of #1 and then #2 covariate feature-groups is shown in Fig 8(d). The first heatmap on the right hand side of information flow reveals two covariate clusters. These two clusters have mixed memberships of three response clusters like the manifestation in the first information flow. The observed entropies with their p-value in parenthesis are calculated as 0.94(0.008) (for green-dominant one) and 0.89(0.019) (for the mixture of blue and orange), respectively.

Through pattern confirmations with small p-values are resulted in both two information flows, the 2nd information flow clearly indicates that extreme small standing and sitting heights are associated particularly with small values of features belonging to #1 feature-group; in contrast large standing or sitting heights are highly associated large values of the same five features in #1 group. Again we demonstrate that these associative patterns are displayed through the linkages between response’s and covariate’s dependency structures. This and the above examples nicely illustrate the exploratory nature of our proposed categorical pattern matching and the resolutions to the issue of multiple response.

The original investigation in [16] was concerned about the issues arising from multicollinearity among the 8 covariate features, including the sitting height, in linear regression analysis with the standing height as the response feature. Again it is rather unnatural that two highly related features: sitting and standing heights, as seen in the Fig 8(A), are separated by the divide between response and covariate. This certainly was done due to the fact of lacking statistical methodology for accommodating Multiple response.

The principle component analysis (PCA) was used to convert the 8 features into a few independent “factors” to alleviate effects of multicollinearity. Again such linearity based artificial factors made the regression results very hard for interpretation. In contrast, our information flow clearly and naturally reveals patterns of response features and links them with associative patterns based on groups of synergistic covariate features with evident heterogeneity.

**Heart disease** This dataset taken from UCI machine learning repository contains 13 features and 270 human subjects. Among 13 features, there are 5 continuous ones: Age(V1), Resting Blood Pressure(V4), Serum Cholestorol(V5), Maximum Heart Rate Achieved(V8), Oldpeak(V10); 3 binary Variables: Sex(V2), Fasting Blood Sugar(V6)(> 120 mg/dl), Exercise Induced Angina(V9); and 5 categorical ones: Chest Pain Type(V3, with values 1, 2, 3, 4), Resting Electrocardiographic results (V7, with values 0, 1, 2), Slope of the Peak Exercise ST segment(V11, with 1: upsloping; 2:flat; 3:downsloping), Number of Major Vessels colored by Fluoroscopy(V12, with 4 values from 0 to 3), thal (V13, with 3 = normal; 6 = fixed defect; 7 = reversable defect), see details in [17]. This example illustrates how to handle digital coding for mixed data types. Data can be found via link:http://archive.ics.uci.edu/ml/datasets/Statlog+%28Heart%29.

Each binary and categorical features are digitally coded for making the digital coding more comparable with continuous features. The coding scheme for a categorical one is based on its closest non-categorical feature. [Digital Coding for binary and categorical features:]

1. **Binary:** {0} → 0 and {1} → 5;
2. **Categorical-V3:** being close to binary V9, {1, 2, 3} → 0; {4} → 5;
3. **Categorical-V7:** being close to continuous V8, {0} → 9; {1} → 3; {2} → 7;
4. **Categorical-V11:** keep ordinal order with {1} → 3; {2} → 6; {3} → 9;
5. **Categorical-V12:** keep ordinal order with {0} → 0; {1} → 3; {2} → 6; {3} → 9;
6. **Categorical-V13:** being close to binary V2, {3} → 0; {6, 7} → 5.

**Fig 9:**
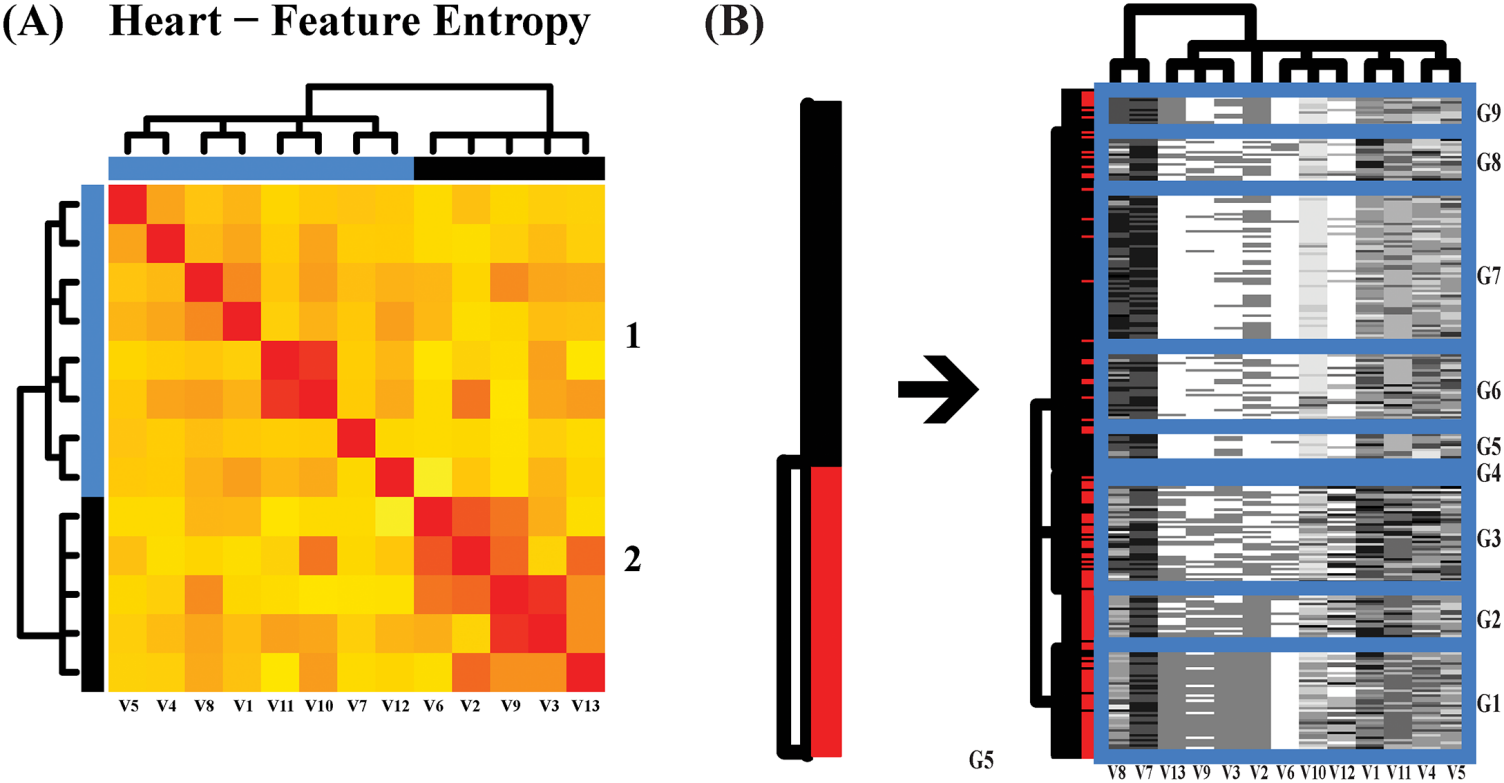
Heatmaps via DM on heart disease data. (a) Mutual entropy matrix of all features with two synergistic groups; (b) Coupling geometries of all features. Red color for patients, Black for healthy subjects.

The mutual conditional-entropy matrix shows two synergistic feature groups in Fig 9(A). The heatmap of involving all covariate features, as shown in Fig 9(B), reveals their joint dependency via two scales of pattered blocks: 1) the fine scale having 9 clusters, denoted by G1 through G9; 2) the coarse scale having 3 conglomerate clusters, (G1, G2), (G3, G4) and (G5, G6, G7, G8, G9). The information flow from response’s patient and healthy subject clusters to involving all covariate features, as shown in Fig 9(B), discovers high degrees of heterogeneity of covariate patterns within the patient as well as healthy subject clusters. It is noted that we also explore information flows based on either of the two synergistic feature-groups. They are not as effective as the one involving with all covariate features.

**Fig 10:**
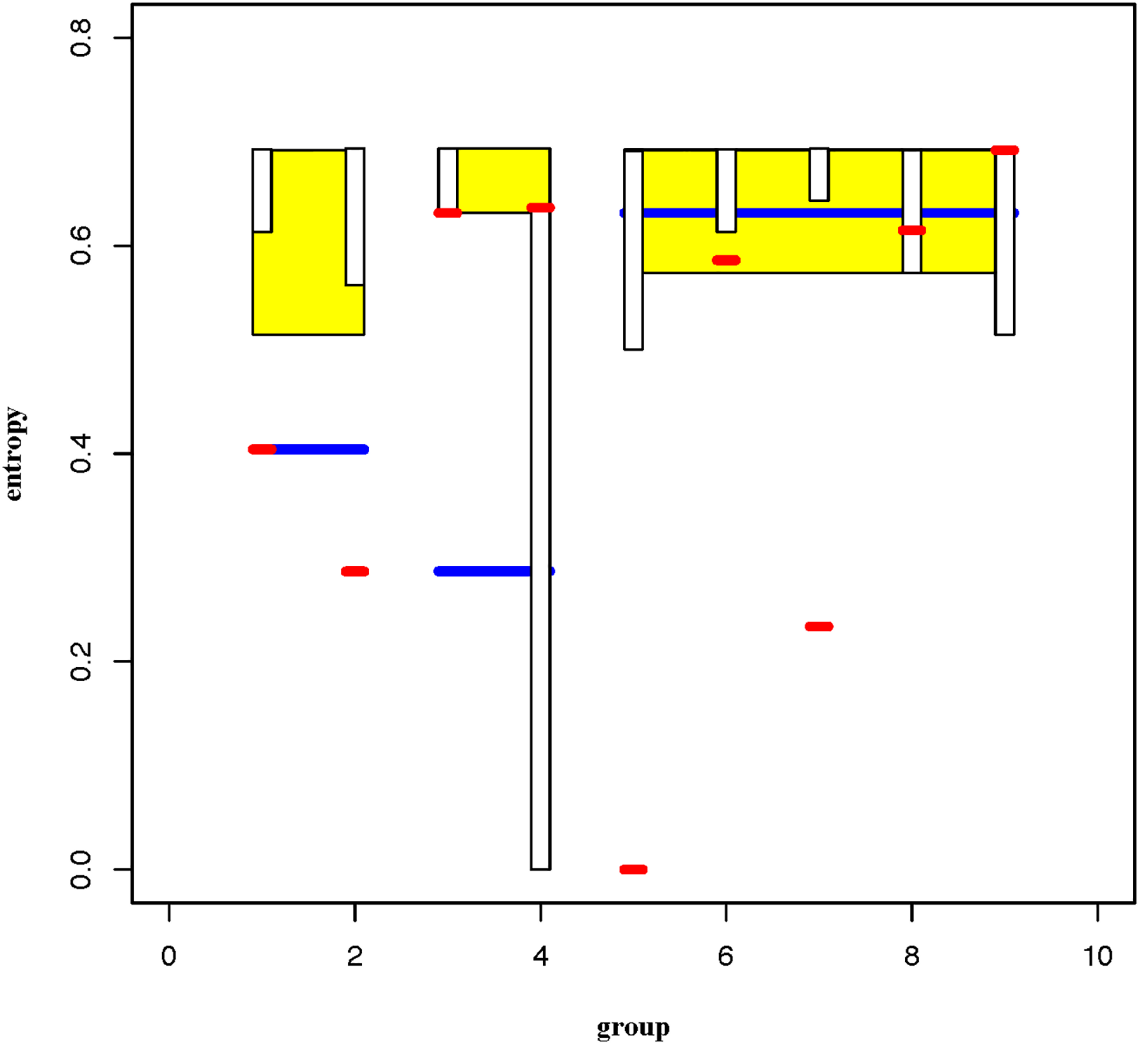
Classification performances of an information flow on two scales. 95% Box-plots of the three-cluster scales is in Yellow color with observed entropy being marked in Blue, while the nine-cluster scale one in Black with observed entropies being marked in Red. The clusters from the left-to-right are arranged exactly to correspond to clusters from bottom-to-top in Fig 9(B). Each box is built based on 1000 simulated entropy values via simple random sampling without replacements.

Further, via simple random sampling without replacement scheme, the classification performance pertaining to two scales of clustering compositions: 3 clusters (Yellow color-coded boxes) and 9 clusters (Black color-coded bars), in the information flow are evaluated through 1000 simulations and presented in box-plots of 95%, as shown in Fig 10. We see that observed entropies (Blue for 3-cluster scale and Red for 9-cluster scale) below their corresponding boxes indicate significant results, that is, the clusters with non-random compositions of patients and healthy subjects with P-values less than 5%. It is noted that a smaller cluster size would render a longer 95% box.

## Conclusion

In this paper we develop one universal platform: algorithmic computing protocol plus graphic display techniques for system data analysis. Our computing protocol is developed under the guiding principle of having multiple synergistic mechanisms contained in a system. Our goal is geared to first extract authentic information contents contained in a system data set. And secondly our categorical-pattern-matching via graphic display is to stimulate proper understanding of computed information, and thirdly to discover pertinent knowledge about the system under study. The resultant system knowledge on one single response mechanism is visible and explainable through a series of covariate mechanisms. Such knowledge is organized and represented through one single information flow. And a system is likely better understood by multiple information flows.

An information flow functionally maps the response’s structural dependency into covariate’s patterned dependency. These dependency patterns and structures are computed through Data Mechanics on data matrix, and are collectively revealed through multiscale blocks framed by a clustering tree superimposed on a group of synergistic features and another clustering tree on the ensemble of subjects. That is, the dependency patterns and structures summarize essential information contents on response and covariate data matrices, respectively, without involving potential distortions possibly caused by unrealistic modeling or distribution assumptions. In this era of big data, we are confident that our universal platform of system data analysis has high potential merits in sciences.

In contrast to our universal platform, it is worth mentioning and discussing the narrow perspective tied to model selection techniques in statistics. On top of employing ad hoc and narrowly focused criterions, like sum of squared error (SSE), all model-selection techniques choose only one set of covariate features from a fixed ensemble of potential models, and ignore all potential groups of features, which might result in a just slightly larger SSE than the minimum one. It seems like that no extra information can be offered from the second best sets of covariate features. This is likely totally not true. Since there might exist several distinct and meaningful mechanisms simultaneously associating with one single dimensional response feature. For instance, consider a study on causes of a behavioral disorder, such as autism or obesity. Wouldn’t the potential causes of a disorder become more and more complex when more than more subjects are included into the study? More subjects certainly will bring in more diverse and different psychological factors, environmental conditions, cultures and genetic makeups and many others. These causal factors surely tightly tangle together as multi-faces of the disorder.

Further all model selection techniques assume an implicit fundamental assumption that the ensemble of potential models is invariant with respect to the number of involving subjects. Like the conditioning argument in all regression analysis, this invariance assumption is another strong evidence of ignorance of structural dependency on the covariate side. To be more specific, as the ensemble of observed system subjects becoming larger, the subject’s “community structure” would be more fully exposed. That is, distinctions and gaps among these communities are to be more evidently expressed through block patterns due to large and fine scales dependency among covariate features. The presence of finer and finer scales dependency structures is exactly the reason underlying the fact that no models are correct when the sample size is really big. But this invariance assumption imposed by all model selection techniques strictly require practitioners to blindly give up the truth that there are potential multiple mechanisms behind trends of one single response feature. On the other hand, this critically unreasonable assumption of fixed ensemble of potential models disregarding sample sizes also reflects the impossibility of the issue of how to properly grow the ensemble as sample size increase.

At the end, we remark that our directed associative linkage expressed through graphic display can fundamentally resolve the recent issue of reproducibility of research results for publications in major scientific journals. The reason is that, even though this reproducibility concern has pressured scientists to be more vigilant and rigorous when they conduct and report their data analysis, unintentional or careless mistakes or human fallacies can still creep into the modeling and affect summarizing parameter values. Requirement of submitting the original data in the submission process for journal publication would only prevent potential human errors to some limited extents. Since effects of man-made assumptions, particularly involved in complicate modeling, are still hard to be filtered out and prevented from contributing to implications made from reported statistical results.

## Supporting information

S1 Box Box for pictorial illustrations of mutual conditional-entropy between two clustering compositions based on two trees. (PDF)

S2 Box Box for formula illustrations of mutual conditional-entropy between two clustering compositions based on two trees. (PDF)

S3 Box Box for illustrating synergistic feature groups through computations of data mechanics. (PDF)
