# Supplementary material for: From patterned response dependency to structured covariate dependency: Entropy based categorical-pattern-matching
Source: PLoS One. 2018 Jun 14;13(6):e0198253. doi: 10.1371/journal.pone.0198253 (PMC6006982; doi:10.1371/journal.pone.0198253)

S1 Box: Diagram of mutual conditional entropy

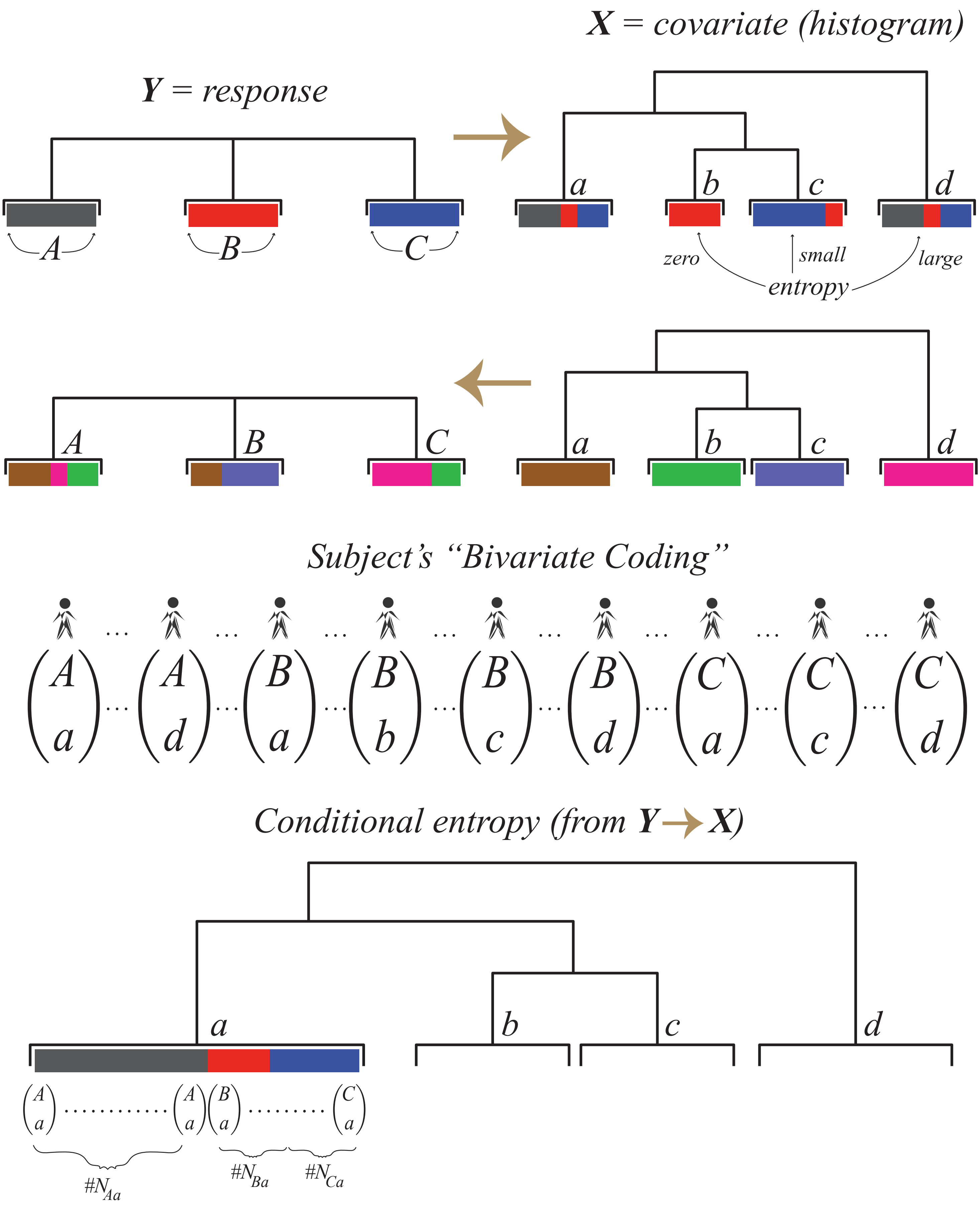

Supplement: S1 Box — (PDF) [file pone.0198253.s001.pdf]
