# Supplementary material for: From patterned response dependency to structured covariate dependency: Entropy based categorical-pattern-matching
Source: PLoS One. 2018 Jun 14;13(6):e0198253. doi: 10.1371/journal.pone.0198253 (PMC6006982; doi:10.1371/journal.pone.0198253)
Supplement: S2 Box — (PDF) [file pone.0198253.s002.pdf]

# S2 Box: Equation of mutual conditional entropy

$$\begin{cases} P_{A|a} = \frac{N_{Aa}}{N_{Aa}+N_{Ba}+N_{Ca}} = P_r[A \mid a] = \{Probability\ of\ Y = A\ if\ X = a\} = P_r[A = Y \mid X = a] \\ P_{B|a} = P_r(Y = B \mid X = a) = \frac{N_{Ba}}{N_{\cdot a}} \\ P_{C|a} = P_r(Y = C \mid X = a) = \frac{N_{Ca}}{N_{\cdot a}} \end{cases}$$

$$N_{\cdot\cdot} = N_{\cdot a} + N_{\cdot b} + N_{\cdot c} + N_{\cdot d}$$

$$W_a = \frac{N_{\cdot a}}{N_{\cdot\cdot}}, W_b = \frac{N_{\cdot b}}{N_{\cdot\cdot}}, \cdots$$

*Conditional entropy given* $X = a$

$$E_a^{(0)}(Y \rightarrow X) = (-1)\{P_{A|a} \cdot \log P_{A|a} + P_{B|a} \cdot \log P_{B|a} + P_{C|a} \cdot \log P_{C|a}\}$$

$$E_a^{(r)}(Y \rightarrow X) = \frac{E_a^{(0)}}{E^{(0)}[Y]}$$

$$with\ E^{(0)}[Y] = entropy\ of\ Y$$

$$E^{(r)}[Y \rightarrow X] = W_a \cdot E_a^{(r)}(Y \rightarrow X) + W_b \cdot E_b^{(r)}(Y \rightarrow X) + W_c \cdot E_c^{(r)}(Y \rightarrow X) + W_d \cdot E_d^{(r)}(Y \rightarrow X)$$

*Mutual (conditional) entropy of* $Y$ *and* $X$

$$E[Y \Longleftrightarrow X] = \frac{(E^{(r)}[Y \rightarrow X] + E^{(r)}[X \rightarrow Y])}{2}$$
