# Supplementary material for: From patterned response dependency to structured covariate dependency: Entropy based categorical-pattern-matching
Source: PLoS One. 2018 Jun 14;13(6):e0198253. doi: 10.1371/journal.pone.0198253 (PMC6006982; doi:10.1371/journal.pone.0198253)

S3 Box: Mutual Conditional Entropy Matrix and Iterative Results of DM

(A)

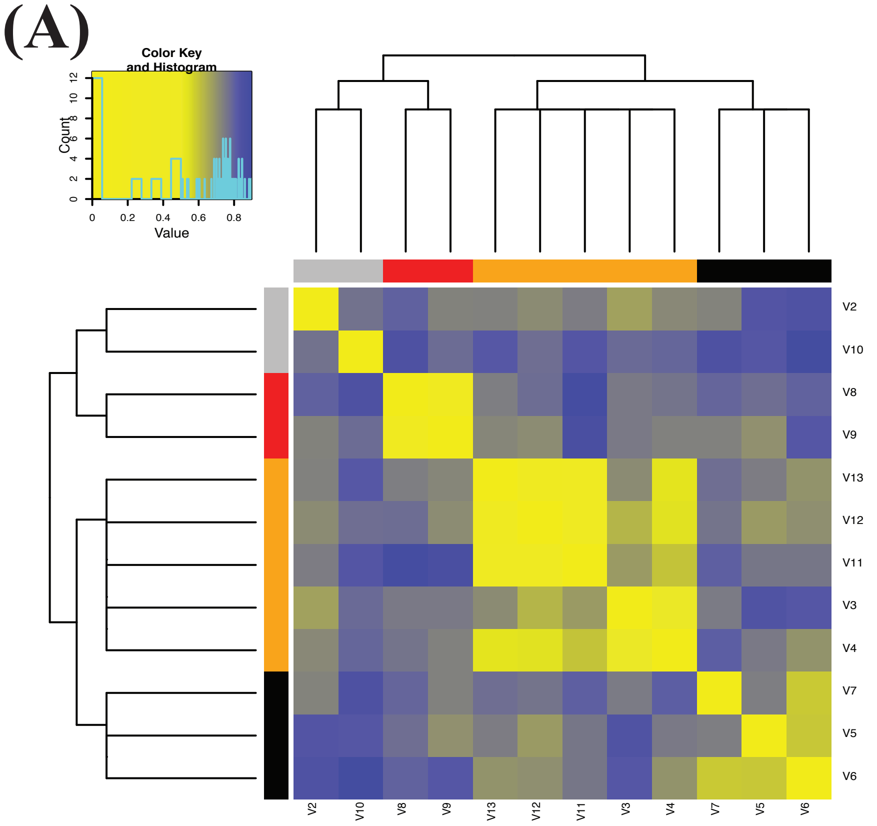

The mutual entropy matrix on the right clearly shows four synergistic feature groups located diagonal. By using the synergistic feature group: G1, the DM computation reveal block patterns in 1st iteration, and improves the result on 2nd iteration.

(B)

1st iteration DM

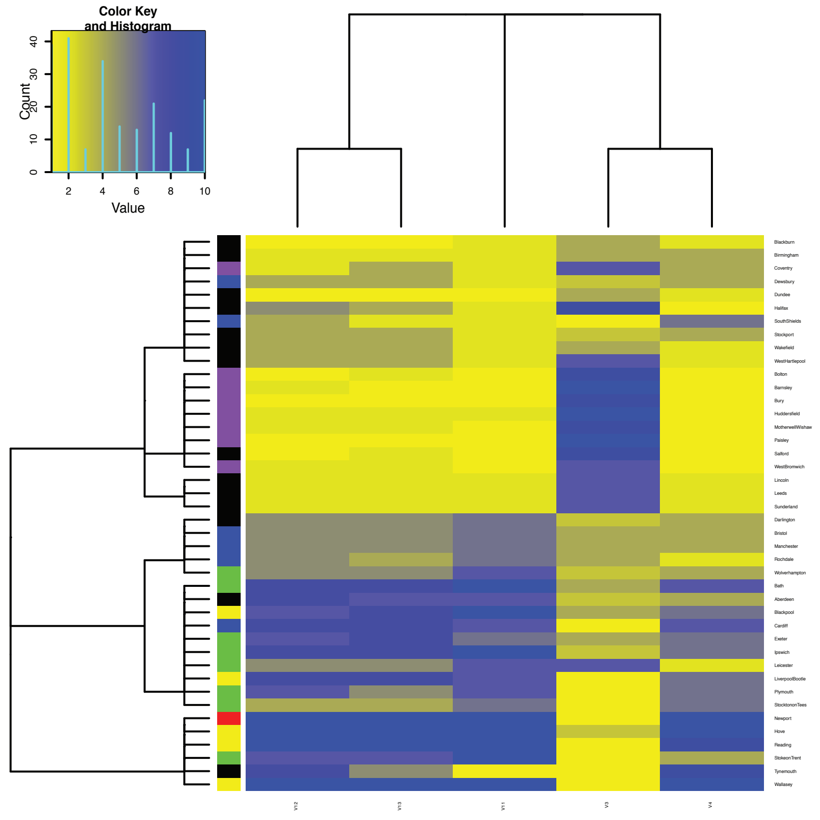

(C)

2nd iteration DM

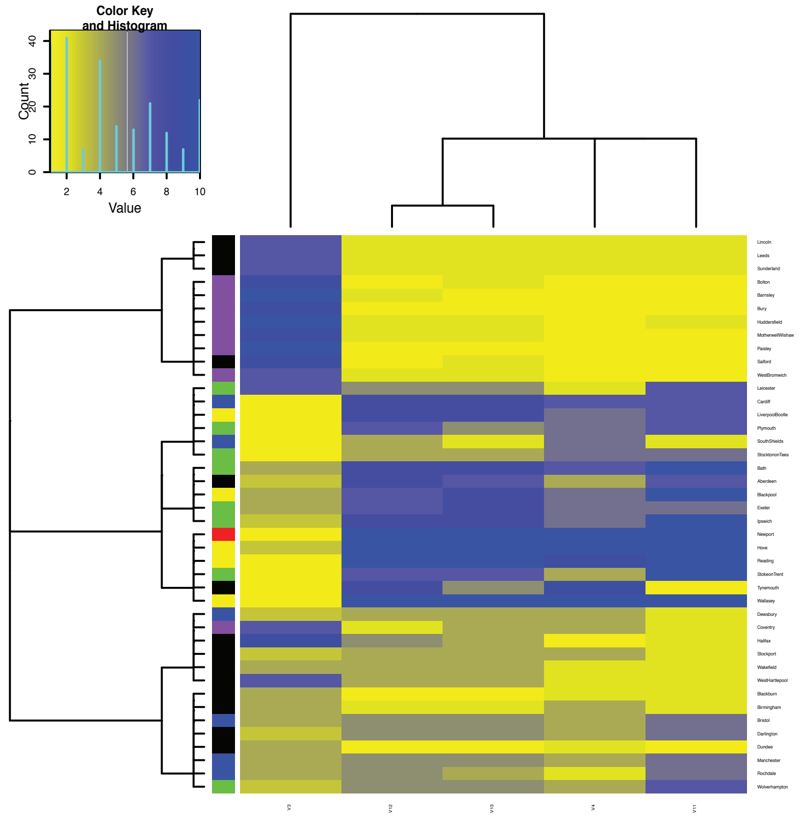

Supplement: S3 Box — (PDF) [file pone.0198253.s003.pdf]
